# Epistatic Analysis of the Contribution of Rabs and Kifs to CATCHR Family Dependent Golgi Organization

**DOI:** 10.3389/fcell.2019.00126

**Published:** 2019-08-02

**Authors:** Shijie Liu, Waqar Majeed, Pranas Grigaitis, Matthew J. Betts, Leslie K. Climer, Vytaute Starkuviene, Brian Storrie

**Affiliations:** ^1^Department of Physiology and Biophysics, University of Arkansas for Medical Sciences, Little Rock, AR, United States; ^2^Centre for Quantitative Analysis of Molecular and Cellular Biosystems (BioQuant), Heidelberg University, Heidelberg, Germany; ^3^Institute of Pharmacology and Molecular Biotechnology (IPMB), Heidelberg University, Heidelberg, Germany; ^4^Institute of Biosciences, Vilnius University Life Sciences Centre, Vilnius, Lithuania

**Keywords:** Golgi analysis, rab, KIF, tether, genetic screen, epistasis analysis

## Abstract

Multisubunit members of the CATCHR family: COG and NRZ complexes, mediate intra-Golgi and Golgi to ER vesicle tethering, respectively. We systematically addressed the genetic and functional interrelationships between Rabs, Kifs, and the retrograde CATCHR family proteins: COG3 and ZW10, which are necessary to maintain the organization of the Golgi complex. We scored the ability of siRNAs targeting 19 Golgi-associated Rab proteins and all 44 human Kifs, microtubule-dependent motor proteins, to suppress CATCHR-dependent Golgi fragmentation in an epistatic fluorescent microscopy-based assay. We found that co-depletion of Rab6A, Rab6A’, Rab27A, Rab39A and two minus-end Kifs, namely KIFC3 and KIF25, suppressed both COG3- and ZW10-depletion-induced Golgi fragmentation. ZW10-dependent Golgi fragmentation was suppressed selectively by a separate set of Rabs: Rab11A, Rab33B and the little characterized Rab29. 10 Kifs were identified as hits in ZW10-depletion-induced Golgi fragmentation, and, in contrast to the double suppressive Kifs, these were predominantly plus-end motors. No Rabs or Kifs selectively suppressed COG3-depletion-induced Golgi fragmentation. Protein-protein interaction network analysis indicated putative direct and indirect links between suppressive Rabs and tether function. Validation of the suppressive hits by EM confirmed a restored organization of the Golgi cisternal stack. Based on these outcomes, we propose a three-way competitive model of Golgi organization in which Rabs, Kifs and tethers modulate sequentially the balance between Golgi-derived vesicle formation, consumption, and off-Golgi transport.

## Introduction

In most mammalian cells, the Golgi apparatus is organized into a dynamic, ribbon-like structure, which is generated by laterally linked Golgi stacks consisting of several cisternae aligned in parallel (for review, see Wei and Seemann, 2010). Over 1000 proteins, mainly discovered in proteomics studies, are known to play a role in biogenesis and maintenance of this organelle (Wu, 2004; Gilchrist et al., 2006; Takatalo et al., 2006). Additional regulators of the Golgi apparatus were identified in large-scale RNA interference (RNAi) microscopy-based screens. This technique is based primarily on visualization and quantitative analysis of Golgi appearance in light micrographs of fluorescently tagged Golgi resident or cargo proteins. Novel regulators of trafficking-related Golgi function have been identified in screens that analyzed Drosophila tER-Golgi units (Kondylis et al., 2011), the morphology of the mammalian Golgi apparatus (Chia et al., 2012), the early secretory pathway (Farhan et al., 2010; Simpson et al., 2012; Millarte et al., 2015), TGN-to-lysosome trafficking (Bickle et al., 2014), or endocytic trafficking (Liberali et al., 2014). In some studies, candidate protein down-regulation or up-regulation by RNAi or cDNA over-expression, respectively, was combined with brefeldin A (BFA) induced redistribution of Golgi complex to ER in order to identify regulators of retrograde Golgi-to-ER trafficking (Lisauskas et al., 2012; Galea and Simpson, 2015). The list of hits derived in these studies identifies several novel individual regulators, but fails to derive their functional interactions. Combinatorial knock-downs provide a solution to this problem and, indeed, Galea and Simpson (2015) demonstrated the synergistic effects between Rab1a and, particularly, Rab1b and a number of other Golgi-related Rabs in controlling retrograde Golgi-ER trafficking.

Because many of the membrane trafficking events associated with the Golgi apparatus occur in the immediate vicinity of the organelle, it is difficult to resolve one from another. However, effects can be more readily determined in the case where the Golgi is scattered in a membrane trafficking-dependent manner. Inhibition/redirection of Golgi membrane trafficking can lead to disruption of the interphase Golgi apparatus with a classic example being the disruption of the Golgi ribbon into scattered mini-stacks due to drug-induced microtubule depolymerization (Rogalski et al., 1984; Thyberg and Moskalewski, 1985; Cole et al., 1996; Yang and Storrie, 1998). Another case is retrograde Golgi tether-dependent membrane trafficking in which tether depletion results in organelle scattering. For instance, COG complex (conserved oligomeric Golgi complex subunit), a member of the CATCHR family (Complex Associated with Tethering Containing Helical Rod) tethers vesicles mediating intra-Golgi membrane trafficking (Lee et al., 2010). The RNAi depletion of the COG3 protein causes Golgi fragmentation accompanied by accumulation of scattered glycosyltransferase-positive vesicles (Zolov and Lupashin, 2005; Shestakova et al., 2007). Knockdown of yet another CATCHR family tether, NRZ complex, through depletion of ZW10 (centromere/kinetochore protein ZW10 homolog) or RINT-1 (Rad50-interacting protein 1) also leads to fragmentation of Golgi ribbon into a cluster of punctate Golgi elements (Hirose et al., 2004; Sun et al., 2007). In interphase cells, ZW10 and RINT-1 mediate retrograde transport between the Golgi apparatus and ER (endoplasmic reticulum) while in mitotic cells the same proteins function to tether the linkage of MAD1 (mitotic spindle assembly checkpoint protein) to chromosomes in a process essential to chromosome segregation (Williams et al., 1992, 1996; Chan et al., 2000; Wainman et al., 2012; Défachelles et al., 2015).

Under the circumstances of double knockdown of tether and a second protein, the suppressive effect of co-depletion becomes a readout to test whether the co-depleted proteins are required for tether-dependent Golgi trafficking. The double knockdown protocol has shown tether-dependent Golgi apparatus organization to be regulated by at least two Rab proteins, Rab6 (Sun et al., 2007; Majeed et al., 2014) and Rab33b (Starr et al., 2010). In double-knockdown experiments, co-depletion of Rab6 strongly inhibited Golgi ribbon fragmentation induced by ZW10 or COG3 knockdown (Sun et al., 2007). Furthermore, of the 15 or more Rab6 effectors, a small subset has been found to be crucial to ZW10 and COG3-dependent Golgi organization. These are motor proteins, MyoIIA or Kif20A, and the dynein motor adaptor, BicD (Majeed et al., 2014). Depletion of BicD by RNAi or overexpression of truncated BicD C-fragment suppresses both ZW10 and COG3-dependent Golgi fragmentation. Here, we designed a phenotype-rescue approach in order to define functional interactions of key Rab proteins and Kifs in tether-dependent Golgi organization. We tested the epistatic effect of RNAi treatments directed against 19 different Golgi associated Rab proteins and all 44 human Kifs. We demonstrated that 4 Rabs and 2 Kifs suppressed both ZW10- and COG3-depletion-induced Golgi fragmentation (ZDI- and CDI-Golgi fragmentation, respectively) strongly indicating that these two pathways share common initial steps. A set of Rab and Kif proteins selectively suppressed ZDI-Golgi fragmentation, whereas, no selective suppressors of CDI-Golgi fragmentation was identified. Based on protein-protein interaction (PPI) network analysis we speculate that the observed results are the outcome of testable direct and indirect protein interactions. These outcomes and approach should be generalizable to large scale studies of protein interactions that define spatiotemporal Golgi organization.

## Materials and Methods

### Cell Culture

HeLa cells stably expressing GalNAcT2-GFP were cultured in DMEM supplemented with 10% fetal bovine serum (FBS) and 0.45 mg/ml Geneticin (Jiang and Storrie, 2005). Cells were grown in a humidified incubator at 37°C and 5% CO_2_. All cell culture media, sera and associated reagents were obtained from Life Technologies, Sigma-Aldrich or Atlas Biologicals.

### Antibodies

Mouse monoclonal β-tubulin antibody was purchased from Sigma-Aldrich. Rabbit polyclonal antibodies were purchased from Abcam (KifC3, Rab27a) and Santa Cruz (Kif25 and Rab6). Mouse monoclonal antibody directed against Rab33B was purchased from the Frontier Institute, Shinko-nishi, Ishikari, Japan (Rab33bd5-Mo-Tk02).

### siRNA Treatment

Both single siRNAs and SMARTPool siRNAs were synthesized by Thermo Fisher Scientific. The accession numbers of all siRNAs are shown in the Supplementary Tables 1, 2. Individual siRNAs targeting specific proteins are indicated by a suffix to the protein name, e.g., -01, -02, etc. For screening experiments, HeLa-GalNAcT2-GFP were grown and transfected in μ-Plates 96 Well (Ibidi). For validation experiments at high numerical aperture, cells were grown and transfected in 35 mm tissue culture dish containing 12 mm diameter glass coverslips. After overnight culture, cells were transfected using DharmaFECT 1 (Thermo Fisher Scientific) according to the manufacturer’s protocol. The next day the transfection was repeated with total incubation post initial transfection being 4 days (Majeed et al., 2014). Similar outcomes were observed at transfection concentrations of either 50 or 100 nM.

### Light Microscopy

For screening, 25 images/well were taken using a Zeiss AxioObserver Z1 inverted microscope fitted with an automated Ludl stage and a Zeiss Definite Focus system. A 20x/0.80 numerical aperture plan apochromat objective magnified to 32× via a 1.6× Optovar was used. All screening experiments were repeated twice. Golgi organization effects as assayed at 32x objective wide field imaging were validated by collection of confocal image stacks with a 63×/1.40 numerical aperture objective and a BD CARV II spinning disk confocal accessory mounted on a Zeiss 200M inverted microscope. Images were processed with iVision-MAC^TM^ software.

### Quantification of Changes in Golgi Organization

Confocal images were first deconvolved using Huygens Professional software to sharpen the distinction between Golgi apparatus and diffuse cytoplasmic fluorescence. To speed image analysis, the deconvolved image stacks were compressed into single plane images using a maximum intensity projection algorithm (MIP, iVision-MAC^TM^ software). The resulting images were then segmented between Golgi apparatus and cytoplasm based on the intensity of GalNAcT2-GFP fluorescence and the number of Golgi fragments and Golgi area determined by segmentation analysis using iVision-MAC^TM^ software. At least 30 cells were analyzed per data point.

### Western Blot and qRT-PCR Analysis of Knockdown Extent

For Western blotting, cells transfected with corresponding siRNA were lysed in hot 2% SDS, followed by standard SDS-PAGE (Sun et al., 2007). Western blotting was performed using primary antibodies and appropriate secondary antibodies conjugated with IRDye 800 dyes (LI-COR). Blots were scanned and analyzed using a LI-COR Odyssey system (LI-COR).

For qRT-PCR analysis of siRNA knockdown, HeLa cells treated sequentially with 100 nM siRNA on day 0 and day 1 and after 4 days total, RNA was isolated using the RNeasy Mini Kit (Qiagen) and 3 μg of RNA was converted to cDNA using the High Capacity cDNA Reverse Transcription Kit (Thermo Fisher Scientific). 100 ng of cDNA template was amplified using Rab-specific primers, or scramble control, via the SYBR^®^ Green PCR Master Mix Kit (Thermo Fisher Scientific). mRNA expression was quantified relative to GAPDH using the ΔΔCT method. Results are reported as the average of 3–4 replicates.

### High-Pressure Freezing, Freeze-Substitution and Electron Microscopy

Cells grown on sapphire disks coated with a 10 nm carbon layer were transfected with siRNA as described above. High-pressure freezing was performed using a Leica EMPACT2 high-pressure freezing unit (Leica Microsystems). 100 mM mannitol and 2% Type IX ultra-low temperature gelling agarose (Sigma-Aldrich) in DPBS supplemented with 2% FBS was used as cryoprotectant. All solutions and sample holders (Swiss Precision Instruments) were pre-warmed to 37°C. All manipulations were carried out on a heating block warmed to 37°C and monitored with a dissecting microscope (Leica Microsystems). Frozen cells were stored in liquid nitrogen.

Specimens were freeze-substituted with anhydrous acetone containing 2% OsO_4_/0.1% glutaraldehyde/1% H_2_O at -90°C for 16–22 h using a Leica AFS unit (Leica Microsystems). Specimens were warmed to 0°C over 2 days, and then moved to the cold room (4°C). In the cold room, the specimens were incubated with acetone containing 1% tannic acid/1% H_2_O for 1 h, then replaced with acetone containing 1% OsO_4_/1% H_2_O and incubated for 1 h. The disks were rinsed repeatedly with acetone before and after each of incubation. After that, samples were warmed to room temperature and then plastic embedded essentially as described previously (Marsh et al., 2001).

50 nm thin sections cut with a Leica UltraCut-UCT microtome were collected and post-stained with aqueous uranyl acetate and Reynold’s lead citrate (Electron Microscopy Sciences) to enhance contrast. Images were taken using a Tecnai F20 intermediate-voltage electron microscope operated at 80 keV (FEI Co.).

### Construction and Analysis of Protein-Protein Interaction (PPI) Networks

We filtered the IntAct database (Orchard et al., 2014) for high-confidence interactions based on biochemical and cell biology assays for physical interactions. High-confidence interactions were then filtered for self-loops, orphaned nodes and redundant interaction pairs. First-degree interactions were visualized with Cytoscape software (Shannon et al., 2003) using an Edge-weighted Force-directed layout and a Prefuse Force-directed layout was used for second-degree interaction networks; both using default weighing schemes. In addition, second-degree interaction partners were also filtered according to their cellular localization (either “Golgi” or “vesicle”) as retrieved from UniProt (Bateman et al., 2017). Scoring of betweenness centrality was used to identify the most crucial elements in respective networks.

## Results and Discussion

### Co-depletion of Rab6 and CATCHR Tethers: Comparative Analysis of Golgi Organization by Fluorescence and Electron Microscopy

**FIGURE 1:**
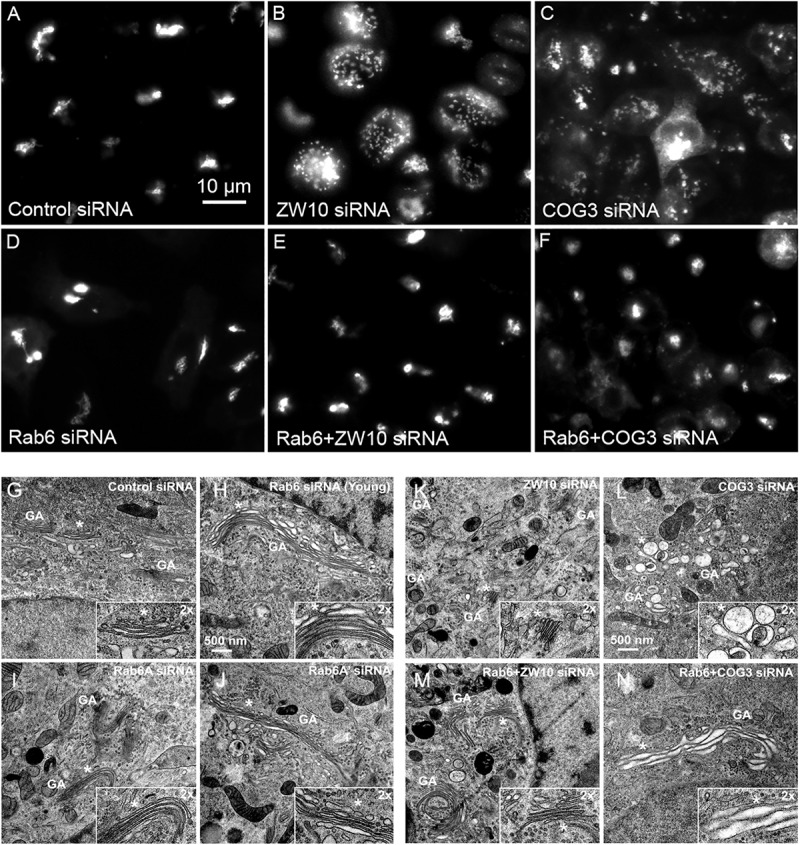
Rab6 and CATCHR protein depletion effects on Golgi organization as revealed by wild field microscopy (32× objective) and EM. HeLa cells stably transfected with GalNAcT2-GFP as a Golgi marker were used in panels **(A–N)**. RNAi treatments were at a concentration of 100 mM as described in Section “Materials and Methods” and cells were processed for microscopy 4 days post initial transfection. siRNAs directed against **(A)** scrambled, Control, **(B)** ZW10, **(C)** COG3. **(D)** Rab6, **(E)** Rab6+ZW10, **(F)** Rab6+COG3, **(G)** scrambled, Control, **(H)** Rab6 (Young), **(I)** Rab6A, **(J)** Rab6A’, **(K)** ZW10, **(L)** COG3, **(M)** Rab6+ZW10, **(N)** Rab6+COG3.

We initially tested strategies for robust quantitative analysis of the Golgi complex when imaged by fluorescence and electron microscopy in three states: native compact, fragmented and restored after the double knockdown of Rab6 and retrograde Golgi tethers. We used stably transfected GalNAcT2-GFP distribution as a marker for Golgi ribbon organization in HeLa cells. In these cells, GalNAcT2-GFP localizes normally to the Golgi apparatus and distributes across the entire juxtanuclear ribbon (Storrie et al., 1998). As expected, in cells transfected with negative control siRNA, the Golgi ribbon was unaffected, presenting as a compact, juxtanuclear Golgi complex in the fluorescence microscope having 4.5 detectable fragments on average (Figure 1A). Consistent with previous results (Storrie et al., 2012), the control Golgi apparatus by EM was composed of closely arrayed cisternal stacks of ∼900 nm in length and containing ∼4 cisternae. There were only a small number of vesicles (∼6 vesicles/stack) associated with the control Golgi apparatus (Figure 1G and Table 1).

**Table 1:** Quantitative analysis of the effect of selected siRNA induced protein depletions on Golgi cisternal dimensions and associated vesicles (thin section electron microscopy).

siRNA	Total number of stacks scored	Average maximum cisternae length (nm) ± SEM	Average number of cisternae per stack ± SEM	Average number of Golgi associated vesicles per stack (within 1 μm of Golgi stack) ± SEM
**Single siRNA**				
Control	11	896.3 ± 120.9	4.0 ± 0.2	6.7 ± 1.1
COG3	ND	ND	ND	ND
ZW10	22	419.3 ± 37.3	3.4 ± 0.2	18.9 ± 2.0
**Rab6**				
Rab6 (Sun)	5	2911.6 ± 394.4	5.6 ± 0.2	57.6 ± 12.5
Rab6 (Young)	7	2718.6 ± 340.0	5.4 ± 0.2	82.4 ± 7.6
Rab6A	8	1577.5 ± 197.1	3.9 ± 0.1	32.8 ± 3.4
Rab6A’	7	1743.9 ± 209.0	4.4 ± 0.2	44.0 ± 5.9
Rab27A	11	1007.9 ± 131.6	3.5 ± 0.2	30.5 ± 3.9
Rab33B	10	1065.3 ± 144.9	3.7 ± 0.3	32.1 ± 3.9
Kif25	12	1023.2 ± 186.8	3.8 ± 0.2	33.1 ± 5.9
KifC3	9	971.1 ± 169.9	3.7 ± 0.2	26.8 ± 4.7
**COG3 + second siRNA (suppression assay)**				
+ Rab6 (Sun)	10	1515.7 ± 361.4	3.8 ± 0.3	36.9 ± 6.4
**ZW10 + second siRNA (suppression assay)**				
+ Rab6 (Sun)	13	856.2 ± 85.1	4.8 ± 0.5	34.5 ± 4.1
+ Rab27A	9	785.6 ± 184.8	3.7 ± 0.2	29.4 ± 2.9
+ Rab33B	12	865.1 ± 115.4	3.9 ± 0.2	22.7 ± 4.1
+ Kif25	15	833.5 ± 141.5	3.7 ± 0.2	21.3 ± 2.5
+ KifC3	9	831.0 ± 87.7	3.8 ± 0.3	25.3 ± 3.1

*ND, not detectable. A stacked Golgi apparatus was not observed in COG3 knockdown cells.*

**FIGURE 2:**
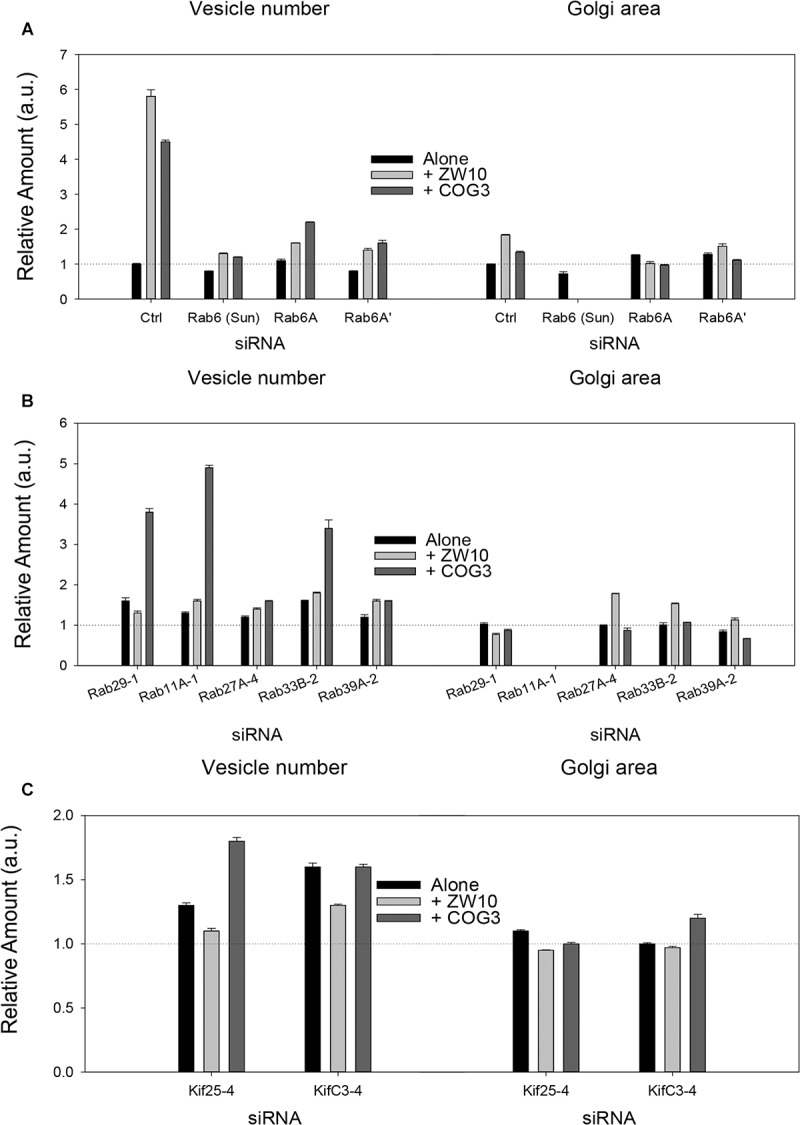
Fluorescence microscopy quantification of Golgi fragmentation and area following RNAi incubation directed against hit Rabs or Kifs. **(A)** Rab6 isoforms, **(B)** Rab hits, **(C)** negative end Kif hits. Quantification of Golgi fragmentation and area was based on the distribution of GalNAcT2-GFP fluorescence. Huygens Professional software was used to deconvolve the images to sharpen the distinction between Golgi apparatus and general cytoplasm. Following deconvolution images were then segmented to determine Golgi fragments and area using iVision for Mac software. At least 30 cells were analyzed per data point and data were normalized to control. The siRNA sequences directed against the proteins labeled on the X-axis are listed in Supplementary Tables 1, 2. The suffix number –1, –2 or –4 following the Rab and Kif corresponds to the siRNA sequence used in the experiment. Bars are plotted ± SEM.

The fluorescent Golgi ribbon in GalNAcT2-GFP cells transfected with ZW10 or COG3 siRNA for 2–4 days was fragmented into clustered punctate Golgi elements that were nearly 5-fold more numerous than control (Figure 1A–C, 2). Under these conditions, the tethers were down-regulated by 80% (Sun et al., 2007; Storrie et al., 2012; Majeed et al., 2014). By electron microscopy (EM), the clustered, perinuclear, dilated vesicles, putative Golgi elements (see. also Zolov and Lupashin, 2005) resulting from depletion of COG3 were larger (Figure 1L) than the small, stacked cisternal elements resulting after ZW10 depletion (Figure 1K). We suggest that in the absence of rapid CATCHR tether-dependent vesicle capture, Golgi-derived vesicles should “wander” producing Golgi ribbon fragments that are detectable even by fluorescence microscopy. By EM, the number of the Golgi-proximal vesicles was increased ∼2-fold with ZW10 siRNA exposure (Figure 1 and Table 1). The maximum diameter of such vesicles was ∼70 nm in cross-sections of control and ZW10 knockdown cells, suggesting that we scored the same vesicle class in both conditions (Supplementary Figure 1).

Rab6 siRNA alone did not change the number of Golgi fragments significantly when analyzed by fluorescence microscopy (Figure 1D, 2). However, by EM, as previously reported (Storrie et al., 2012), significant structural alterations can be observed: the number of Golgi cisternae increased by 1 to 2 per stack, cisternal length increased by about 3-4-fold, and the number of Golgi-associated vesicles increased nearly 10-fold (Figure 1H and Table 1). Less striking effects were observed when Rab6A and Rab6A’ isoforms were depleted individually (Figure 1I,J). Rab6 protein levels as antibody detected were higher, 40% versus 25% (Supplementary Table 3A). By EM, Rab6A and Rab6A’ knockdowns individually produced an elongate Golgi stack, about half the size of the Rab6 knockdown, with ∼half the vesicle accumulation of the Rab6 knockdown and no increase in cisternal number (Figure 1I,J and Table 1). Interestingly, the frequency of U-shaped Golgi stacks was high with Rab6A knockdown, but not Rab6A’ and what appear to be tubular-vesicular carriers were detected in the Rab6A knockdown (Figure 1H). In sum, by EM the two isoforms produced less strong effects and differed somewhat in their phenotypic consequences. Interestingly, by fluorescence microscopy, Golgi area was increased by ∼25% when RNAi depletion of Rab6 is performed in an isoform-dependent manner (Figure 2), but is more compact (by 25%) when both isoforms were depleted. We suggest that this outcome is consistent with the higher Rab6 protein levels and indicates that a single Rab6 isoform can support sufficient trafficking to lead to modest Golgi expansion. Our data show, that Rab6 variants recruit factors needed for normal trafficking through the Golgi apparatus and/or off Golgi trafficking.

We have previously demonstrated by fluorescence microscopy that RNAi-mediated down-regulation of Rab6 efficiently rescues fragmentation of the Golgi complex, caused by the depletion of both retrograde ZW10 and COG3 tethers (Sun et al., 2007; Majeed et al., 2014) and, as shown in (Figure 1E,F), this result is obvious at the resolution of a 32x objective screening magnification. Furthermore, by EM, we now tested for the first time whether Golgi cisternal organization was restored. We found that the extent of restoration was more complete for ZW10 than COG3 depletion (Figure 1M,N). Using our standard protocol in which siRNAs targeting the tethers and either the Rab6 A or A’ isoform were transfected simultaneously at equimolar concentrations, we could now show that both isoforms rescued tether-induced fragmentation. However, in contrast to Rab6 co-depletion, downregulation of Rab6A and Rab6A’ separately restored ZDI-Golgi fragmentation slightly more efficiently than that of CDI-fragmentation in terms of the number of the Golgi fragments visible by fluorescence microscopy (Figure 2). By EM, we demonstrate that suppression resulted in a normalization of Golgi cisternae length accompanied by a decrease in the number of Golgi proximal vesicles (Figure 1M,N and Table 1) suggestive of a rebalancing of membrane trafficking toward an organized Golgi cisternal stack. Interestingly, in the case of ZW10 co-depletion, vesicle size was skewed to a slightly larger diameter (Supplementary Figure 1) and the number of cisternae displayed across the Golgi stack frequently varied across the length of the stack (Figure 1M). In the control Golgi stack, cisternal length is fairly constant going across the stack while, in the ZW10-/Rab6 co-depletion case, a group of cisternae may extend only a 1/3rd of the way along the length of the stack giving the approach of a shorter stack piggy backing on top of a longer stack, one that contains fewer cisternae (Figure 1M).

### Combinatorial RNAi-Mediated Screen of Rab Proteins Involved in CATCHR Mediated Maintenance of the Intact Golgi Complex

We next applied epistatic RNAi-based experimentation to screen Rab GTPases in retrograde tether-dependent Golgi trafficking pathways. We tested the epistatic rescue effect of siRNAs directed against 19 Golgi-related Rab proteins chosen on the basis of literature evidence (e.g., Goud et al., 2018; Supplementary Table 1). The microscopy-based primary screen was performed in a wide-field modus with a 32x objective configuration, 0.80 numerical aperture, and the hits then validated by confocal microscopy with a 63× objective (see section “Materials and Methods”).

Eight out of 19 chosen Rabs were knock-downed by four individual siRNAs and Rab4A was targeted with 2 individual siRNAs (Supplementary Table 1). The remaining eight Rabs (Rab1A, Rab1B, Rab2A, Rab10, Rab14, Rab30, Rab34, and Rab43) were targeted with one siRNA only that had been validated in previous studies. Depletions were confirmed by antibody blotting or by qRT-PCR (Supplementary Table 3B) as appropriate. As expected, Rab1A and Rab2A induced a strong fragmentation of the Golgi complex when depleted (Supplementary Table 1 and Table 2). None of the other Rabs targeted by a single siRNA impaired the Golgi complex in a fluorescence microscopy read-out or rescued both ZDI- and CDI-fragmentation.

**Table 2:** Summary of Rabs and Kifs knockdown screening against ZW10 or COG3 knockdown.

Basal effect on Golgi apparatus		Effect on Golgi fragmentation	
**Rabs**			
Fragment Golgi apparatus (2)	Rab1A, Rab2A	Suppress both ZW10 and COG3 KD (6)	Rab6, Rab6A, Rab6A’, Rab22A, Rab27A, Rab39A
No effect alone (18)	Rab1B, Rab4A, Rab6, Rab6A, Rab6A’, Rab29, Rab8A, Rab10, Rab11A, Rab14, Rab22A, Rab27A, Rab30, Rab33B, Rab34, Rab38, Rab39A, Rab43,	Suppress ZW10 KD only (3)	Rab29, Rab11A, Rab33B
		Suppress COG3 KD only (0)	None
		Non-suppressive (9)	Rab1B, Rab4A, Rab8A, Rab10, Rab14, Rab30, Rab34, Rab38, Rab43
**Kifs**			
Fragment Golgi apparatus (10)	Kif4A, Kif10, Kif11, Kif12, Kif14, Kif15, Kif21A, Kif24, Kif26A, Kif26B	Suppress both ZW10 and COG3 KD (2)	Kif25, KifC3
Stretched strands of Golgi apparatus (2)	Kif20A, Kif23	Suppress ZW10 KD only (10)	Kif2C, Kif3A, Kif4B, Kif5A, Kif6, Kif7, Kif9, Kif14, Kif17, KifC2
Toxic to HeLa cells (1)	Kif18A	Suppress COG3 KD only (0)	None
No effect alone (31)	Kif1A, Kif1B, Kif1C, Kif2A, Kif2B, Kif2C, Kif3A, Kif3B, Kif3C, Kif4B, Kif5A, Kif5B, Kif5C, Kif6, Kif7, Kif9, Kif13A, Kif13B, Kif16A, Kif16B, Kif17, Kif18B, Kif19, Kif20B, Kif21B, Kif22, Kif27, KifC1, KifC2, KifC3, Kif25	Non-suppressive (23)	Kif1B, Kif1C, Kif2A, Kif2B, Kif3B, Kif5B, Kif5C, Kif10, Kif11, Kif12, Kif13B, Kif16A, Kif16B, Kif18A, Kif18B, Kif19, Kif20B, Kif21A, Kif21B, Kif24, Kif26A, Kif26B, Kif27

*Rabs highlighted in red were further tested by electron microscopy. Kifs highlighted in red were further tested by electron microscopy.*

All suppressive phenotype hits were found among Rabs targeted by multiple siRNAs. A Rab was considered as a suppressor when a strong rescue phenotype was achieved with 50% of siRNAs or a weak rescue was obtained by three out of four siRNAs. Six Rabs – Rab11A, Rab22A, Rab27A, Rab29, Rab33B, and Rab39A were suppressive for ZDI-fragmentation. For Rab27A and Rab39A, at least one siRNA out of four also showed the suppression for CDI-fragmentation in both replicates of the primary screen (Supplementary Table 1). We note that, as previously shown (Starr et al., 2010), siRNA directed against Rab33B at a high concentration (200 nM) suppressed Golgi fragmentation induced by COG3 knockdown. The latter effect was not reproduced with the much lower concentrations of siRNAs used in our assay, which turned out to be sufficient to cause more than 60% of the protein depletion (Supplementary Table 3B). Therefore, we scored Rab33B as a suppressor of ZDI-fragmentation only. None of the other Rabs were selective strong suppressors of CDI-fragmentation only.

**FIGURE 3:**
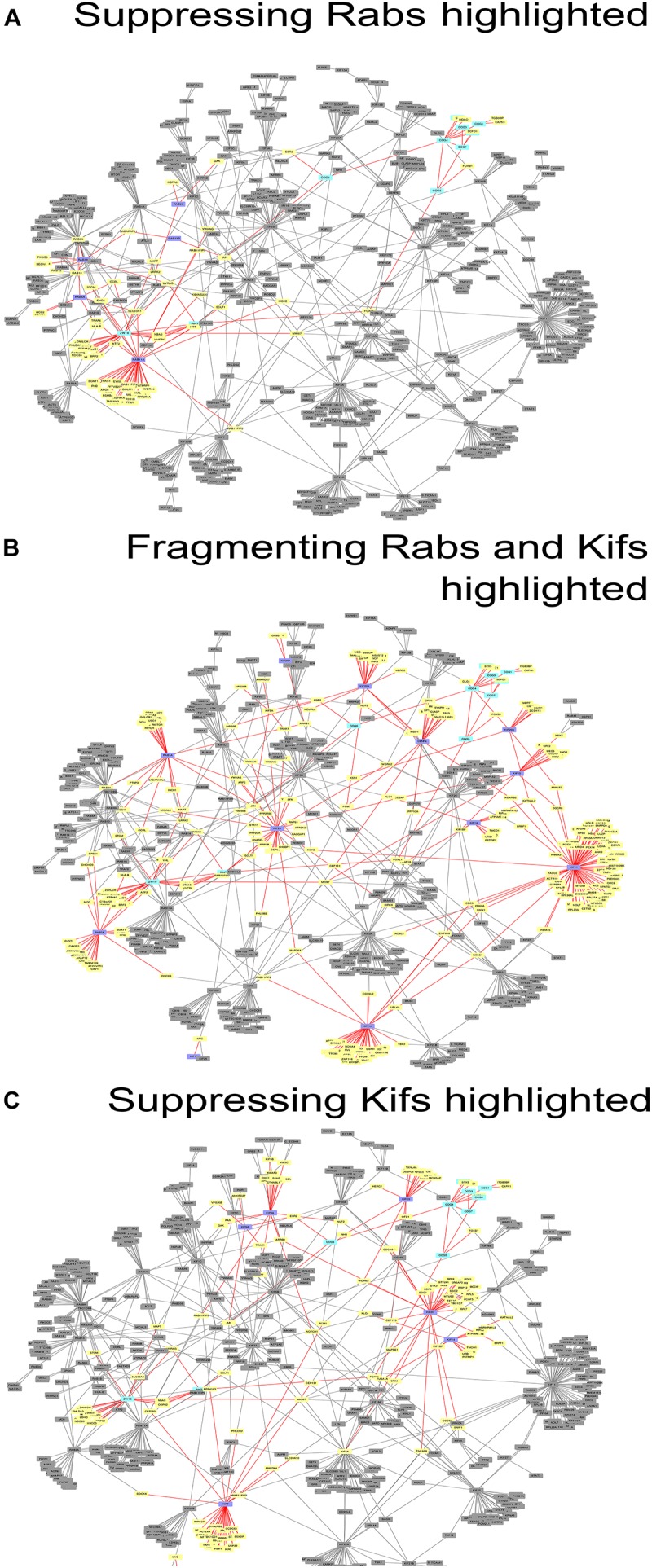
Networks of direct protein-protein interactions with highlighted Golgi fragmentation-suppressing Rabs **(A)**, Golgi-fragmenting Rabs and Kifs **(B)** and Golgi fragmentation-suppressing Kifs **(C)**. Protein-protein interaction networks were constructed as described in Materials and Methods. Color code for the nodes: purple, hit Rabs and Kifs; yellow, direct interactors of the screening hits; cyan, proteins of tethering complexes; gray, proteins not forming direct interactions with the seed nodes. Edges colored in red represent direct interactions the seed nodes (suppressing Rabs, Golgi-fragmenting Rabs and Kifs or suppressing Kifs for **(A–C)**, respectively) possess. See also Supplementary Table 5.

These data were also reflected in the PPI network (Figure 3) we constructed, using the tethers, Rabs and Kifs screened in this study (comprising 723 nodes, interconnected by 886 edges) and the tethers, effector Rabs and their direct interactors (99 nodes and 110 edges). All five hit Rabs present in the network were experimentally shown to suppress ZDI-fragmentation, but none of them were a direct interactor with this tether complex in the PPI network. Instead, they all are positioned closer to ZW10 as indicated by the lesser number of edges, required to connect Rabs with ZW10/RINT proteins, compared to the subunits of COG complex.

Interestingly, four out of seven suppressing Rabs (Rab6, Rab11A, Rab33B and Rab39A) are direct interactors to each other in the PPI network, suggesting a cooperative, non-redundant Rab-mediated control of Golgi organization. Two other Rabs, Rab8A and Rab10, also scored in the top ten with respect to their betweenness centrality value in the network analysis, (Figure 3A and Supplementary Figure 2A). However, these did not score as hits in our experiments. Based on this analysis, we suggest that the literature-guided choice of a single Rab10 siRNA should be widened. On the other hand, Rab8A which was depleted with four siRNAs failed to be included, because the two weak hits did not consistently replicate (Supplementary Table 1). We also observed that double knock-down of Rab2A and the tethers aggravated the fragmentation of the Golgi apparatus. However, that was not observed in the case of RNAi targeted against Rab1A that acts one step upstream from Rab2A. Possibly, unique interaction partners of these two spatially related, but sequentially acting Rabs produce the observed difference (Supplementary Figure 3A).

As indicated by the analysis of a second-degree interaction network for the screened Rabs (Supplementary Figure 3), the rescue of CDI-fragmentation by Rab27A and Rab39A may be an indirect effect. Additionally, the analysis suggests that indirect interactions through a BECN1-dependent ZW10-regulatory pathway (Frémont et al., 2013) may explain the differential effects of siRNA targeting Rab39A. Rab39A depletion induced reduction of the Golgi area, which was further aggravated by co-transfection with siRNA, targeting COG3 (Figure 2B). In contrast, the rescue of ZW10 fragmentation by Rab39A downregulation was complete in terms of area and numbers of the Golgi fragments at the level of fluorescence.

**FIGURE 4:**
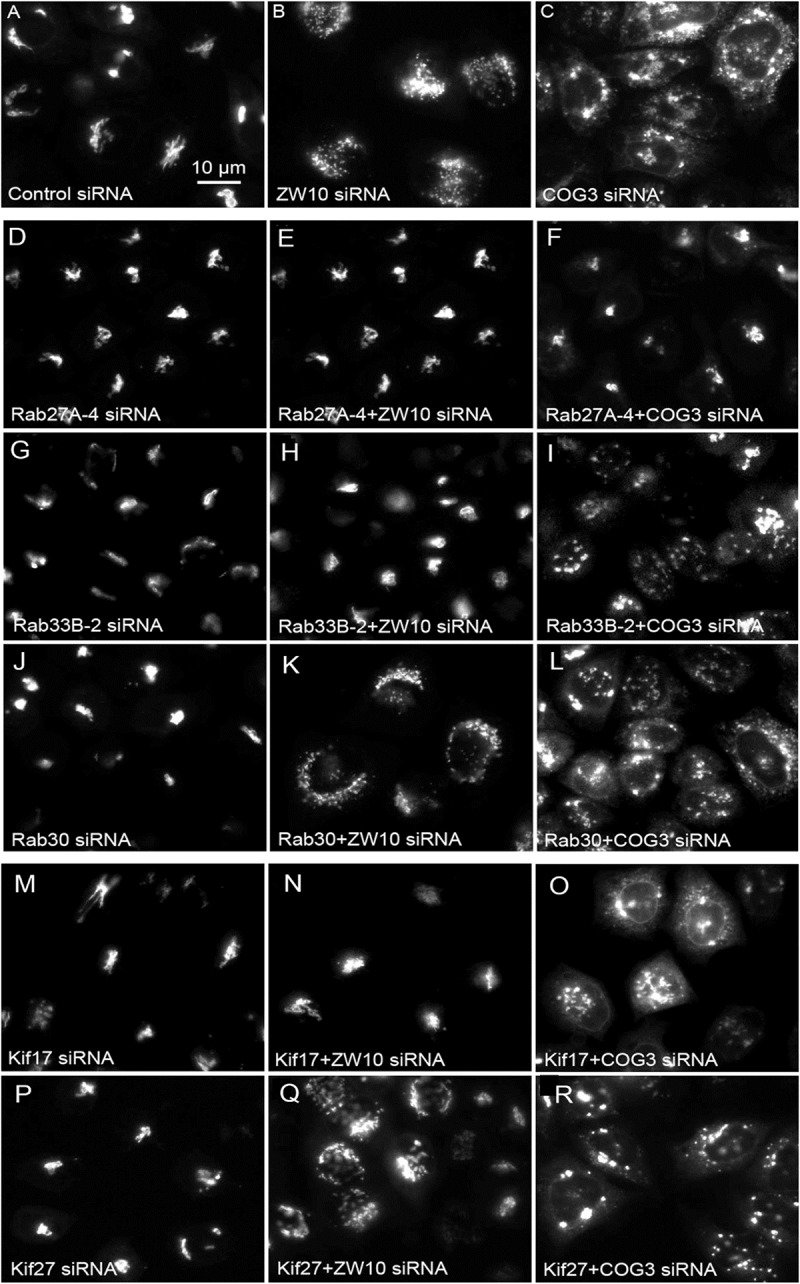
Wide field microscopy (32× objective) revealed suppressive effects of Rab or Kif co-depletion on CATCHR protein-dependent Golgi fragmentation. Reference single siRNA treatments: **(A)** siControl, **(B)** siZW10, and **(C)** siCOG2. **(D–L)** Tests of the suppressive effects of various Rab siRNAs: **(D–I)** Rab27A and Rab33B as positive examples of suppression of Golgi fragmentation and **(J–L)** Rab30 as a negative example of suppression. **(M–R)** Tests of the suppressive effects of Kif siRNA SMARTPool examples.

For the majority of our hits (Figure 4A–L and Table 2), we provide the first evidence associating them with the retrograde Golgi trafficking. Only Rab11A and Rab2A along with our positive control Rab6 were previously shown to act in the retrograde trafficking of Golgi membranes to the ER upon addition of BFA (Galea and Simpson, 2015). Interestingly, the Rabs called as hits in both studies are evolutionary conserved from yeast to humans (Pereira-Leal and Seabra, 2001), indicating the role of these regulators in the most basic and, possibly, ancient routes of membrane trafficking. In contrast, human specific proteins (Rab22A and Rab29) or Rabs expressed in higher eukaryotes (Rab27A, Rab33B and Rab39A) were identified only in our study focused on the specific CATCHR-dependent retrograde trafficking, possibly indicating evolutionary co-evolvement of these Rabs and tether proteins.

### Validation of Rab27A and Rab33B as Regulators of CATCHR Mediated Post-cisternal Golgi Trafficking

**FIGURE 5:**
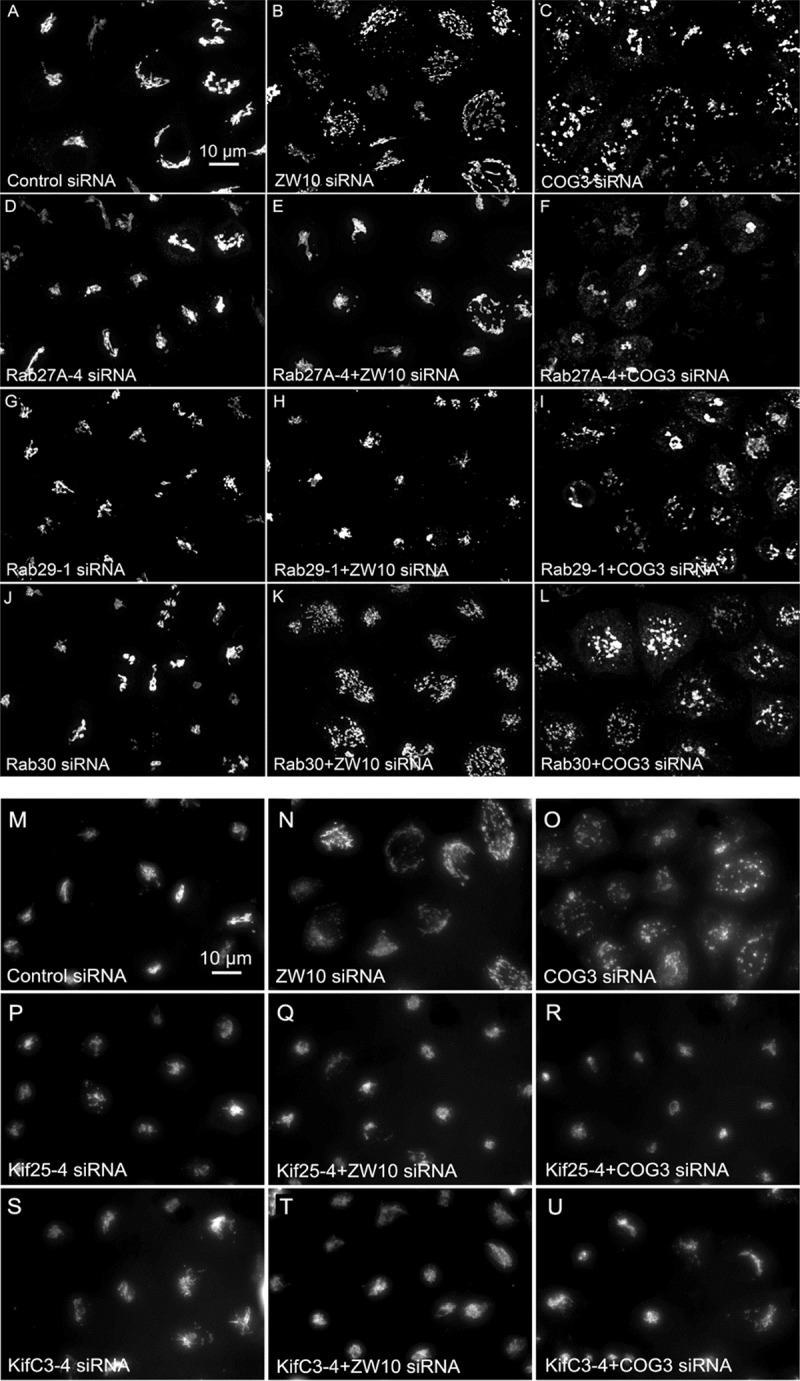
**(A–L)** Comparative suppressive ability of Rab protein directed siRNAs as validated by confocal microscopy (multiplane, maximum intensity projection images). Rab30 siRNA, negative example. **(M–U)** Suppressive effect of negative end motor protein directed siRNAs on Golgi fragmentation (wide field microscopy, 32× objective).

**FIGURE 6:**
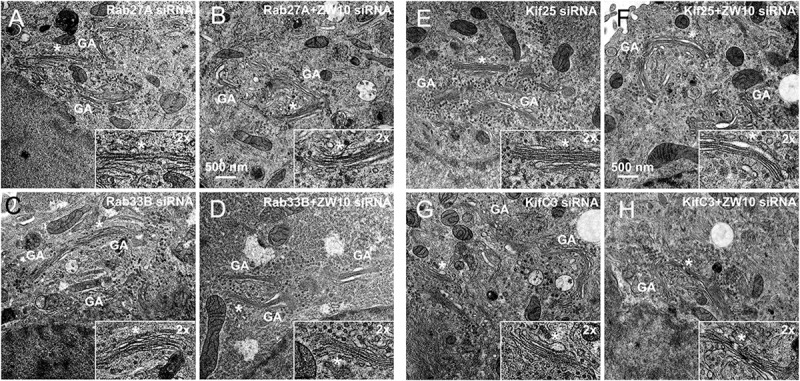
EM validation of the suppressive effects of siRNAs directed against Rab27A **(A,B)**, Rab33B **(C,D)**, Kif25 **(E,F)**, and KifC3 **(G,H)** on CATCHR-protein dependent Golgi fragmentation.

We next repeated the assay with the siRNAs that showed the strongest suppression effect and acquired the images in a confocal modus. In all cases, the phenotypic data were reproduced (Figure 5A–L). All effector Rabs induced a little fragmentation of the Golgi complex (by a factor of 1.2–1.6) when observed by the confocal microscopy. We selected Rab27A and Rab33B, in particular, for EM studies as cases representing dual tether- and ZW10 only-specific suppressors, respectively. Rab27A is a non-Golgi protein, but its role in the transport of Golgi-derived vesicles was recently shown (Stinchcombe et al., 2001; Handley et al., 2007; Bello-Morales et al., 2012; Matsunaga et al., 2017). Furthermore, the efficient RNAi-mediated downregulation of them both could be tested on a protein level (Supplementary Table 3B and Supplementary Figures 4C–F). The knockdown of these two Rabs caused little increase in the cisternal length (Table 1), in contrast to fairly large changes observed by RNAi of Rab6. Nonetheless, the impairment of Golgi trafficking was obvious by 5-fold increase of the Golgi-proximal vesicles (Figure 6A,C and Table 1). Knockdown of Rabs may lead to local vesicle accumulation that limits Golgi fragmentation. Our data suggest, that the depletion of Rab27A and Rab33B impairs off-cisternal/post-cisternal effects, like release of the vesicles. In contrast, Rab6 may be necessary for the organization of cisternae itself as indicated by longer cisternal lengths in depletion experiments.

Rab27A and Rab33B similarly reconstituted the normal length of the cisternae in the double knockdowns. In case of Rab33B, nearly 30% less vesicles were also observed in the double knockdown (Table 1 and Figure 6B,D). All in all, close to normal cisternal organization was restored and at the same time there was a decrease in the number of Golgi associated vesicles, suggesting a rebalancing of vesicle transport under these conditions toward normal Golgi cisternae organization. It is likely that Rab knockdowns suppress the fragmentation by inhibiting motor recruitment.

### Kif Proteins Involved in CATCHR-Dependent and Independent Golgi Organization

We have previously shown, that depletion of a known Rab6 effector, Kif20A, suppresses tether-induced Golgi fragmentation (Majeed et al., 2014). Here, we expanded the screen and the hit scoring procedure with the aim of determining all Kifs that play a role in CATCHR-mediated Golgi trafficking events. SMARTPool siRNAs directed against all 44 known human Kifs were screened and changes in GalNAcT2-GFP distribution analyzed visually (Supplementary Table 2). As a first step, we tested which of the Kifs altered the organization of the Golgi complex when down-regulated alone. Incubation of individual SMARTPools directed against 10 Kifs fragmented the Golgi apparatus into clustered punctuated Golgi elements in the interphase cells suggesting that the vesicle trafficking in these cells was altered (Table 2). A few of these (e.g., Kif12) enhanced Golgi fragmentation in the epistatic assay, similar to Rab2A. The relatively large number of Kifs fragmenting the Golgi when depleted may reflect the overlapping activities of the motors. Partial crosstalk between the individual SMARTPool siRNAs can be ruled out as multiple sequence database comparisons between siRNAs and the corresponding mRNA sequences failed to show overlaps in siRNA sequences. Nine Kifs showed little to no effect in the epistatic experiments and only Kif14 selectively suppressed ZW10-induced fragmentation (Table 2). All 10 Golgi fragmenting Kifs are plus-end motors (Hirokawa et al., 2009). Finally, Kif20A and Kif23 inhibited cytokinesis and thus caused the accumulation of multinucleate cells and long networked strands of Golgi apparatus. SMARTpool directed against Kif18A was toxic to HeLa cells.

Of the 12 Kifs that were effective suppressors of tether-dependent Golgi fragmentation in either a ZW10- or COG3-knockdown background (Supplementary Table 2, Table 2, and Figure 4M–R), 10 were plus end directed motors. The double suppressors were Kif25 and KifC3, both minus-end motors (Noda et al., 2001) that have common binding partners at the centrosomes (e.g., CEP170, WDR62), (Supplementary Figure 2B). A third minus-end motor, KIFC2, showed the suppression of COG3-induced fragmentation in one of two replicates (Supplementary Table 2) and hence may deserve further consideration. Rescue effects were tested further by using four individual siRNAs/gene. Of the four individual siRNAs directed against Kif25 and KifC3, two were suppressive, and caused 60–70% decrease of the respective protein expression (Figure 5M–U and Supplementary Figures 4G–J). The best performing siRNAs were used for EM studies. Similar to the suppressive Rabs, Kif25 and KifC3 shifted the morphology to the Golgi complex toward the normal state (Table 2 and Figure 6E–H). Furthermore, not only the vesicle numbers, but also the distance of these vesicles from the Golgi cisternae were reduced in double ZW10/Kif knockdowns by ∼30% as compared with ZW10 depletion only (Supplementary Table 1). As with Rab6 co-depletion, a slight increase in vesicle diameter was observed (Supplementary Figure 1). KifC3 was previously shown to be necessary for Golgi positioning and maintenance (Xu et al., 2002). In contrast to the depletion of other minus-end motors, like cytoplasmic dynein (Harada et al., 1998; Majeed et al., 2014) or KifC1 (She et al., 2017), no fragmentation of the Golgi complex was observed in our study as well as under the conditions of KifC3 being completely removed by targeted promoter trapping strategy under normal levels of cholesterol (Xu et al., 2002). That may reflect the varying input of each Kif for Golgi positioning or their different expression levels in different experimental systems. Also, our data suggest an intriguing possibility, that minus-end motors that do or do not fragment the Golgi complex when depleted may interact with different subpopulations of microtubules (MTs), namely, centrosome-derived and Golgi-derived MTs, respectively (Chabin-Brion et al., 2001; Zhu and Kaverina, 2013).

Protein-protein interaction network analysis provided little further evidence on the role of these Kifs (Figure 3C). In contrast to Rabs (Figure 3A), no clear PPI distribution pattern and/or closer positioning (in terms of the degree of interaction) of Kifs to ZW10 in the respective PPI network was observed: the suppressive Kifs were quite evenly distributed over the whole PPI network. On the other hand, evolutionary tree comparison was more informative. Notably, nearly all suppressive Kifs were distributed within families 1, 2, 4, 5, 6, 9 of the Kif evolutionary tree (Hirokawa et al., 2009) and largely clustered away from the evolutionary tree families containing Golgi-fragmenting Kifs, distributed over the families 3, 7, 8, 10, 11, 12, 13, 14. Few exceptions were nevertheless found: e.g., Kif11 caused Golgi fragmentation when downregulated, but belongs to the family 5, which is more typical for the suppressive Kifs. The clustering of Kifs into two evolutionary based groups may reflect processivity and, thus, spatial labor distribution among these molecular motors, that drive vesicle release, anchor newly formed vesicle, and mediate off-stack vesicle trafficking. Kifs, that are able to travel over long distances rapidly, would propel vesicles away from the organelle. For instance, KIF3A operates over long distances between the Golgi and ER when mediating retrograde COPI vesicle trafficking (Stauber et al., 2006). Similarly, Kif5A is proposed to regulate motility of nearly 50% of Golgi-derived vesicles (Woźniak and Allan, 2006) and is characterized by a remarkably high processivity (Toprak et al., 2009; Milic et al., 2014). Both of these Kifs were measured to move with the velocity of 600–800 nm/sec (Svoboda et al., 1993; Guo et al., 2019). Consequently, RNAi of such Kifs should induce vesicle accumulation around the Golgi cisternae and little changes to the overall Golgi morphology. The effect is maintained if capturing of the vesicles by the tether is impaired – we suggest that could be an explanation of a suppression phenotype. The picture is different when Kifs having lower velocity (100 nm/sec) are depleted, such as Kif11 (Guo et al., 2018, 2019) or loaded Kif15 that readily detaches from MTs under these conditions (McHugh et al., 2018). Then, the balance is shifted toward the distribution of vesicles away from the Golgi complex, likely, along the tracts of microtubules, followed by Golgi reassembly at distant sites (“fragmentation phenotype”). Consequently, little chance of suppressing loss-of-tether phenotype occurs in this situation.

## Conclusion

**FIGURE 7:**
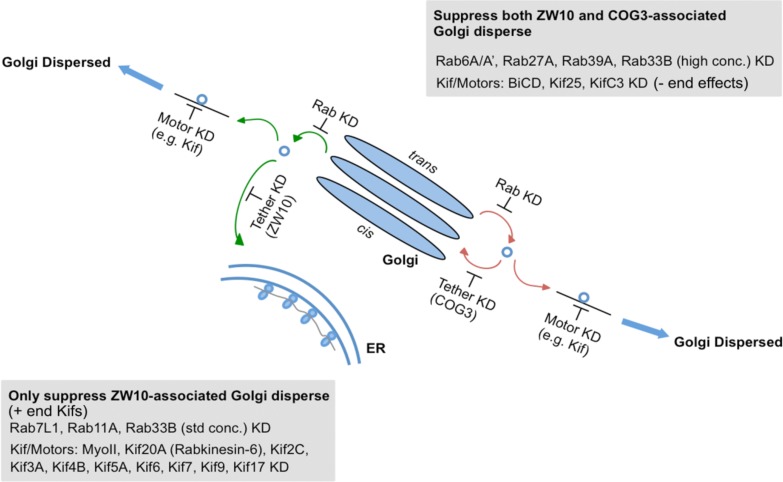
A working model for a selective competition, tug-of-war, between tethers, Rabs, and motors in determining Golgi apparatus organization.

As summarized in Figure 7 and Supplementary Table 4, we have identified Rabs and Kifs in an epistatic retrograde tether-dependent visual assay that are essential for the proper cisternal organization of the Golgi complex. We validated the screening hits via electron microscopy and propose a model of interactions among Rabs, Kifs and tether protein complexes to maintain a morphologically intact Golgi complex. Our data suggest that interphase Golgi apparatus is dynamically unstable due to a tug-of-war between retrograde tether proteins and Kifs acting at long- and short-range distances. The role of the tether would be as a timer or switch ensuring that on-stack vesicle consumption is normally quick. Notably, parallel single and double knockdowns of Kifs and the tethers in our study enabled us to distinguish between tether-dependent and -independent groups of Kifs - information that was not available before. Finally, these studies provide a starting point for future detailed mechanistic experiments to characterize the functional links between individual Rabs and Kifs as well as their redundant or singular activities in spatiotemporally determining Golgi organization.

## Data Availability

All datasets generated for this study are included in the manuscript and/or the Supplementary Files.

## Author Contributions

SL, WM, PG, MB, and LC performed the experiments, data analysis, figure and table preparation, and materials and methods portions of the manuscript. VS and BS directed the experiments, analyzed the data, prepared most of the manuscript, and edited earlier versions of the manuscript.

## Conflict of Interest Statement

The authors declare that the research was conducted in the absence of any commercial or financial relationships that could be construed as a potential conflict of interest.
