# Supplementary material for: Epistatic Analysis of the Contribution of Rabs and Kifs to CATCHR Family Dependent Golgi Organization
Source: Front Cell Dev Biol. 2019 Aug 2;7:126. doi: 10.3389/fcell.2019.00126 (PMC6687757; doi:10.3389/fcell.2019.00126)
Supplement: Supplementary file 1 [file Data_Sheet_1.pdf]

Supplementary Material for

Epistatic Analysis of the Contribution of Rabs and Kifs to CATCHR Family Dependent Golgi Organization

Shijie Liu[1], Waqar Majeed[1], Pranas Grigaitis, Matthew J. Betts, Leslie K. Climer, Vytaute Starkuviene[2], and Brian Storrie[2]

[1]Co-first authors in alphabetical order

[2]Co-senior authors

Supplemental Table 1. Summary of Rab screening against ZW10 and COG3 using single siRNA.

siRNA treatment		Golgi phenotype					
		Experiment 1			Experiment 2		
		Alone	+siZW10	+siCOG3	Alone	+siZW10	+siCOG3
Control		C	-	-	C	-	-
ZW10		F	-	-	F	-	-
COG3		F	-	-	F	-	-
**Single siRNA (100 nM)**							
**siRNA treatment**	**siRNA sequence**	**Experiment 1**			**Experiment 2**		
Rab1A	GTCCAGCATGAATCCCGAATA	F	F	F	F	F	F
Rab1B	AAGATCCGAACCATCGAGCTG	C	F	F	C	WS	F
Rab2A	GCGUACGCCUAUCUCUUCAAGUACA	F	F	EF	F	EF	EF
Rab4A-1	ACGGCCAUGUCCGAAACCUACGAUU	C	F	F	C	F	F
Rab4A-2	GAUGCAGAUCGUGAAGUUACCUUCU	C	F	WS	C-LF	F	F
Rab6-Sun	GAGAAGAUAUGAUUGACAU(UU)	C	S	S	C	S	S
Rab6-Young	GACATCTTTGATCACCAGAd(TT)	C	S	S	C	S	S
Rab6A-Young	GAGCGGTTCAGGAGCTTGAd(TT)	C	S	S	C	S	S
Rab6A'-Young	AGGCTTCAGCTGTGGGATAd(CT)	C	S	S	C	S	S
Rab8A-1	GAAUUAAACUGCAGAUAUG	C	F	F	C	F	F
Rab8A-2	GAACAAGUGUGAUGUGAAU	C	WS	F	C	WS	F
Rab8A-3	GAACUGGAUUCGCAACAUU	C-LF	F	F	C-LF	WS	F
Rab8A-4	GAAGACCUGUGUCCUGUUC	C	F	F	C	F	F
Rab10	ACCTGCGTCCTTTTTCGTTTT	C	F	F	C-LF	F	F
Rab11A-1	GUAGGUGCCUUAUUGGUUU	C	S	F	C	S	F
Rab11A-2	GCAACAAUGUGGUUCCUAU	C	S	F	C	S	F
Rab11A-3	CAAGAGCGAUAUCGAGCUA	C	S	F	C	S	WS
Rab11A-4	GGAGUAGAGUUUGCAACAA	F	F	F	F	F	F
Rab14	CAACUACUCUUACAUCUUU	C	F	F	C	F	F
Rab22A-1	GUAGGUAAAUCGAGUAUUG	C	WS	F	C	WS	F
Rab22A-2	GGACUACGCCGACUCUAUU	C	WS	F	C	WS	F
Rab22A-3	CCUUAGCACCAAUGUACUA	F	F	EF	F	F	F
Rab22A-4	CGCGAUAAACAUAAAUGAA	C	WS	WS	C	WS	WS
Rab27A-1	GAGCAAAGUUUCCUCAAUG	C	WS	F	C	WS	F
Rab27A-2	GGAGAGGUUUCGUAGCUUA	C	WS	F	C	F	WS
Rab27A-3	CCAGUGUACUUUACCAAUA	C	S	F	C	S	F
Rab27A-4	CGACAGCGUUCUUCAGAGA	C	S	WS	C	S	S
Rab29-1	CAGAUUGACCGGUUCAGUA	C	S	F	C	S	F
Rab29-2	GAGAACGGUUUCACAGGUU	C	WS	WS	C	WS	WS
Rab29-3	GGGACUACAUCAAUCUACA	C	S	F	C	WS	F
Rab29-4	CAACAAGUGUGAUCUGUCC	C	WS	F	C	F	F

Rab30	AAGAGAGATTTCGGTCCATTA	C	F	F	C	F	F
Rab33B-1	CACAAACCAUUAAUGCUUA	C-LF	F	F	C	F	F
Rab33B-2	GAUAGAAGAAUGCAAACAA	C	S	F	C	S	F
Rab33B-3	GAUAUACCACGGAUUCUUG	C	WS	F	C	S	F
Rab33B-4	GGUGGAUUUCCGAGAACGA	C	F	F	C	F	F
Rab34	CCTTCAGTCTCCAACTTTG	C	F	F	C	F	F
Rab38-1	GCAAAUGAGUGUGACCUAA	C	WS	F	C	WS	F
Rab38-2	UAUCGCAGGUCAAGAAAGA	C	F	F	C	WS-F	F
Rab38-3	GGAGCACGGUUUCGUAGGA	C	F	F	C	F	WS
Rab38-4	GAGAAGCYAUGGGUGCAUU	C	F	F	C	F	F
Rab39A-1	UGAUUUAGCUUCACAACGU	F	F	F	F	F	F
Rab39A-2	CAGGAGCGGUUCAGAUCAA	C	S	S	C	S	S
Rab39A-3	GUGAAAGAUUGGCUAGAAG	C	F	F	C	F	F
Rab39A-4	AAUCUUGACGAGAGACAUA	C-F	S	F	C-F	S	F
Rab43	GCGTCGACTTCACCATGAA	C-LF	F	F	F	F	F

Rab siRNAs suppressing both ZW10 and COG3 knockdown are highlighted in yellow; Rab siRNAs suppressing ZW10 knockdown only are highlighted in turquoise.

Abbreviations:
C- Compact
WS - Weak Suppression
S- Suppression
LF- Light Fragmentation
F- Fragmentation
EF- Enhanced Fragmentation

Supplementary Table 2. Summary of Kif screening against ZW10 and COG3 using Kif SMARTPool siRNAs.

siRNA treatment		Golgi Phenotype					
		Experiment 1			Experiment 2		
		Alone	+siZW10	+siCOG3	Alone	+siZW10	+siCOG3
Control		C	-	-	C	-	-
ZW10		F	-	-	F	-	-
COG3		F	-	-	F	-	-
SMARTPool (100 nM, 25 nM individual siRNA)							
Plus-end Kifs							
siRNA treatment	siRNA sequences	Experiment 1			Experiment 2		
Kif1A	GGACAGAGCGGGAGUGUGA<br>GAAGAAGACAGAUUUCAUU<br>CAGCAUCUCUGCCGAAUAU<br>GUACCGAGAUUCCGUGUUG	C	W<br>S	F	C	W<br>S	F
Kif1B	CAAACUGGUUCGUGAAUUA<br>GCUCAGAAACCUACGUAAA<br>GCCAAAGGGACUCGAUUAA<br>GGGAGUUGCCAUUCGGGAA	C	F	F	C	F	F
Kif1C	GAACCAGAGUGCUCAGCUA<br>GCAAUGCCAUCAUCAACGA<br>GCAAGUGUAUCGGGACAUU<br>GCAGCAAGGCAUCGACAUA	C	F	F	C	F	F
Kif2A	GAAAUGGUUUACAGGUUUA<br>GGAAUGGCAUCCUGUGAAA<br>UAGAGGAAGUUUGGAUUAU<br>GCGCAGCGAUGGCCGAAUA	C	F	F	C	F	F
Kif2B	GGGAAUAGCUGUCGGACUU<br>GAAGGGAUCUAGCCAAUGG<br>CAGCAAACCUUGUCUGAUG<br>CGAAAUGGGUUGCGAUGAU	C	F	F	C	F	F
Kif2C	GAAUUUGCCCGAAUGAUUA<br>GGAAACGCCCACUGAAUAA<br>GGAAGUGGCAAGACACAUA<br>GGGCUACUUUGGAAUGUCA	C	S	F	C	S	W<br>S
Kif3A	AAACAAAGCUCGACAUGGA<br>UAUCGUAACUCUAAACUGA<br>ACACAAAGGUUAGAGGUUA<br>GCGAUAAUGUGAAGGUUGU	C	S	F	C	S	F
Kif3B	GGUCAGGGCUUCUUACUUA<br>GCAGAAACGUCGAGAAAGA<br>GAGAGUAAGUUGCUUGUUG<br>GAAGAUCAUUGGAAACUAC	C	F	F	C	F	F
Kif3C	GAAUUAGGAUUUCAAAGUG<br>GUACAGGGCUGAAAACAUA<br>UCAAGUACCUAAUCAUCGA<br>ACAAGACCCUCGUGCGCUA	C	W<br>S	F	C	W<br>S	F

Kif4A	GGUAAUAGCCAUACUCUUA<br>GCAAUUGAUUACCCAGUUA<br>GAAAGAUCCUGGCUCAAGA<br>UAAAAGAACGAGACCGUAA	F	W<br>S	F	F	W<br>S	F
Kif4B	GGAAUUGGCUUGGAAAUGA<br>AGAAACAUCUUCCUAAUGA<br>GGUAACAGCCACACUCUUA<br>GCAAGAUCCUGAAAGAGAU	C	S	F	C	S	F
Kif5A	GUUGUGAGCUUCCUAAAUU<br>GAAAUCAAAUCGUCAUGUG<br>CAAAGAAUAUCAACAAGUC<br>CGACUAUGUUCAUCUGUUG	C	S	F	C	S	F
Kif5B	GCAGUCAGGUCAAAGAAUA<br>GAACUGGCAUGAUAGAUGA<br>CAACAGACAUGUAGCAGUU<br>GCAGGAACGUCUAAGAGUA	C	F	F	C	F	F
Kif5C	GAUCUGACCACCCGAGUUA<br>GGUUAUAACGGGACGAUUU<br>GCAAAUUAUUGGAACGGAU<br>CUGGAUAACUGCCGAAGUA	C	F	F	C	F	F
Kif6	GGAACUGCAUGACAACUAU<br>UAUAGAAAGCUACGAGAUA<br>GAAUCUUGAUGAGUCUAUA<br>GCGGAUAUGCGUAAAGUUC	C	VS	F	C	VS	F
Kif7	GAAAUAGGAUCAGCAACUG<br>GAACUGCGGCUGCGGUUAG<br>GAGCUGGUCCGCACAGGAA<br>GGAUGAUUGAUGUCCGGAA	C	S	F	C	S	F
Kif9	UGGAGAAGCUGCCCAGUUA<br>GGACACUGGACGAGAUCGA<br>GUAGUGAGAUCAACCGAAU<br>CAAUAUGGUCCUCGUGACA	C	S	F	C	VS	F
Kif10	CAACAAAGCUACUAAAUCA<br>GGAAAGAAGUGCUACCAUA<br>GGAAAGAAAUGACACAGUU<br>GAACUAAGAAGAAGCGUAU	F	F	F	F	F	F
Kif11	GCAGAAAUCUAAGGAUAUA<br>CAACAAGGAUGAAGUCUAU<br>CAGCAGAAAUCUAAGGAUA<br>CUAGAUGGCUUUCUCAGUA	F	F	F	F	F	F
Kif12	ACAGCAAGCUCACCAAGUU<br>GAUCUACAAUGAGCAGGUU<br>UGGCAGAGAUGUUGACGGA<br>GGUCUCAGCCGUCGAAGGA	F	EF	F	F	EF	F
Kif13A	GAACCAUGCUGUUGUGAAU<br>AGACGUACAUUGAGAAGUA<br>GCAAAUUUGUGCCUUAUCG<br>AAAUAAGUCUCGAACGGUA	C	W<br>S	F	C	W<br>S	F
Kif13B	GAUAAAUGCUUCCUUGUGA<br>GUACUUGGAUCAACAAUUG<br>GAAUAUCUCUUCAGUCUUC<br>GCAAACAAGGUUAUUCUUA	C,<br>LF	F	F	C,<br>LF	F	F
Kif14<br>Multinucleate	GAAUGAAGAUCCACAACUA<br>GGAAUUAUGUUUCAAAUGG<br>AGAAACACGUCUUACAUUA	F	S	F	F	S	EF

	GAAUAAAGGUGUACCAAAG						
Kif15	GAAGAGAGCUUGCUUGCUA<br>GGACAUAAAUUGCAAAUAC<br>GGGAAGACAUUUACUAUGA<br>AAUCAGAGCUUCAGUCUUU	F	W<br>S	F	F	W<br>S	F
Kif16A	CCAAGUAGCUGAACACGUA<br>CCAAACCACUGACGAGUUA<br>GGAGUCAUAUUGCUUAGUU<br>CCACAAUUCUAGCGUAUCA	C,<br>LF	F	F	C,<br>LF	F	F
Kif16B	GAACCUGUCUGCAGUCAUG<br>GGAGAAACCCUAAGUACUC<br>CGUGAAGUCUGCAUUUGAA<br>AGACAUACGUUGGUAGAGA	C	F	F	C	F	F
Kif17	GGAAGAGCCUCACACAUCA<br>GCAACUACUUCCGAUCUAA<br>CCACAUCCCGUCAUCACAA<br>GAGAAGAUCGAUUACUUGG	C	VS	W	C	VS	F
Kif18A	GCAAAGAACUUCAGCCUAU<br>UCAAAGAGAUCGAACAUUU<br>GGAGGAAACUGUCAAACUA<br>GCUAUCAGCUCAAACAUAA	FC	F	F	C	F	F
Kif18B	GGGCAAAGACCUGACGUUU<br>ACCAGGAGGUGUAUAAUGA<br>UGACCCGCCUGCUCAAAGA<br>CUCGGGAGCUGGACAGUCA	C	F	F	C	F	F,<br>FC
Kif19A	GCAAUGACAUGGAGUAUGA<br>GAAAGGAGUGCUACGCUAA<br>CCAAGAACAUUAAGACUAG<br>GAACGUCUCCUACCACAUC	C	F	F	C	F	F
Kif20A<br>Multinucleate	GCAAGAACCUGCUAUCAGA<br>GAAGAGGGCCAGAAGAAUA<br>GCAAUGAGAUGGUAGAACA<br>CUGAAGAGUUGCAUAAGUA	SH	SH	EF	SH	SH	EF
Kif20B	UGAAAUGUCUCGUGUAAUU<br>GAAGAAAUCACACAGUUAA<br>GGAAGAAUGUAAAGAGAUU<br>CCUAAAGUCUCAUAUGGAA	C	F	EF	C	F	F
Kif21A	GGGAAGAUAUUGCCAGUAA<br>GGAAGAAAGUCAAGAAGUG<br>GAGCAGAUCUACAUUCAAU<br>GGUAGAAUCUUACUCAGAA	F	F	F	F	F	F
Kif21B	CCAAGGACCACUACGUUAA<br>UCGAGGAGCUACGGACUAA<br>CGGGUAAAGUUGUGGAAUU<br>CGACCUGACUUGUGAGAUC	C	F	F	C	F	F
Kif22	GGACAUCUAUGCAGGUUCA<br>CAAGCUCACUCGCCUAUUG<br>GCUGGUCGCUGUCGGCUAA<br>GAACAGCGGGAGCAAGUGA	C	W<br>S	F	C	W<br>S	F
Kif23<br>Multinucleate	CGACAUAACUUACGACAAA<br>GACAUGAUCUUUAACAGUA<br>GAACAAAUCACUAUAAGUC<br>GCAUAGAAGUGAUCAAUAA	SH	SH	EF	SH	SH	EF
KIf24	GGAACACCCUGGAGAAUAG<br>GUUCUGAAAUCCAAGUGUA	F	F	F	F	F	F

	GAGUUGAGCUCUCCUUUGG AAUCAGAGUUUGUGUUCGA						
Kif26A	CGACGCAGCUCCCACAUGU GGUCUGGCGUGCGUCAGUA UGGAGUGUCUCGCGGGUCA GAGCCAAGCAUGUGCCGUA	LF	F	F	F	LF	F
Kif26B	GCAAACACAUUCCAUACAA GCAACCAGCUUCAGUGGGA CGGACAGCCUCUCCUAUUA GGGAACAUGAACUGCCGUA	F	F	F	F	F	F
Kif27	GAAGAUGGAUCAUGGUAUU AGAGAGAGCUUAAGAAAUG CCAAACAGCCAGCAAGUUA GGGAUAAUAUGGUUCGUGA	C	F	F	C	F	F
Minus-end Kifs							
KifC1	GGACUUAAAGGGUCAGUUA GUGCUAAGAUGCUCAUGUU GGAGCUCACUGUCACCAAU UGACCUAAAUGCAGAACUA	C	W S	F	C	W S	F
KifC2	AGAAAGCCCUUCCCACUUC GAAAAGGGUUCAGCAUCUG GAGGAGGAUCAGAGGGCGU CCGCCAAGUGGUUACCCAA	C	S	S	C	S	F
KifC3	GUAAGAAGUGCCACAAUGA CUGCGGAGAUCUACAAUGA GGUCACCUCUUGCAUUGAU ACAAUGAGCUCGUGCGGCU	C	S	S	C	S	VS
Kif25	GGAAGAGGAGGCCGGAUUG CAAGAAGCCCAAAGGUUGA CCUACUCACUUCUCUCUUG CGAAGUUACUGGUGAUUCU	C	S	S	C	S	S

Kifs suppressing both ZW10 and COG3 knockdown are highlighted in yellow; Kifs suppressing ZW10 knockdown only are highlighted in turquoise.

Abbreviations:
C- Compact
WS - Weak Suppression
S- Suppression
VS- Very Strong Suppression
LF- Light Fragmentation
F- Fragmentation
EF- Enhanced Fragmentation
FC- Few Cells
SH- Stretched Hands

Supplemental Table 3. Treatment of HeLa cells with siRNAs directed against Rab6, Rab27A and Rab33B effectively knockdown each Rab mRNA and protein level.

A. Effect of siRNA treatment on Rab6 isoform protein expression

Single siRNA	Relative mRNA expression (mean ± SD, % of the control)	Relative protein amount (normalized to β-tubulin)
Control	100.0	1.00
Rab6A	ND	0.41
Rab6A'	ND	0.44
Rab6 (Young)	ND	0.24
Rab6 (Sun)	ND	0.27

B. qRT-PCR quantification of siRNA treatment on selected Rab mRNA levels or protein expression

Rab27A-4	2.2 ± 1.8	0.34
Rab30-1	7.3 ± 1.4	ND
Rab33B-2	34.2 ± 12.8	0.37
Rab34-1	55.8 ± 32.7	ND
Rab34-2	66.8 ± 19.2	ND
Rab43-1	14.0 ± 3.3	ND

4 day siRNA treatment at 100 nM. Averages are quoted plus and minus the Standard Deviation of the Mean. ND, not determined

Supplemental Table 4. UniProt/PubMed Summaries for Rab/Kif Screening Outcomes against ZW10 and COG3

Protein name	UniProtKB Accession No.	General function information from UniProt	Golgi related function information from PubMed[1]
(A) Rabs			
**Suppress both ZW10 and COG3 knockdown**			
Rab6a	P20340	Protein transport. Regulator of membrane traffic from the Golgi apparatus towards the endoplasmic reticulum (ER). Has a low GTPase activity.	They generally collectively referred to as Rab6 because of the high degree of biochemical and phenotypic similarity. Rab6 is involved in retrograde transport through the Golgi apparatus (Utskarpen et al., 2006; Young et al., 2005), and essential for the maintenance of Golgi ribbon organization and vesicle trafficking (Sun et al., 2007; Majeed et al., 2014).
Rab6a'	NA[2]	NA	
Rab27a	P51159	Plays a role in cytotoxic granule exocytosis in lymphocytes. Required for both granule maturation and granule docking and priming at the immunologic synapse.	Required for regulated secretion in cytotoxic T lymphocytes (Stinchcombe et al., 2001). Present in the Golgi and Golgi-derived proacrosomal vesicles in wild-type and Hrb mutant mouse spermatids and involved in vesicle transport (Kierszenbaum et al., 2004). Associated preferentially with newly synthesized granules and is
Rab39a	Q14964	Plays a role in the maturation and acidification of phagosomes that engulf pathogens, such as S.aureus and M.tuberculosis. Plays a role in vesicular trafficking. Plays a role in the fusion of phagosomes with lysosomes. Negatively regulates LPS-induced autophagosome formation in macrophages possibly by implicating PI3K (PubMed:24349490). May be involved in multiple neurite formation (By similarity).	A Golgi-associated protein involved in endocytosis of HeLa cells (Chen et al., 2003).
**Suppress ZW10 knockdown only**			
Rab29/7L-1	O14966	Rab GTPase key regulator in vesicle trafficking. Essential for maintaining the integrity of the endosome-trans-Golgi network structure. Together with LRRK2, plays a role in the retrograde trafficking pathway for recycling proteins, such as mannose 6 phosphate receptor (M6PR), between lysosomes and the Golgi apparatus in a retromer-dependent manner. Regulates neuronal process morphology in	Essential for maintaining the integrity of the *trans*-Golgi network (TGN) and participates in the retrograde trafficking of M6PRs (Wang et al., 2014).

		the intact central nervous system (CNS). May play a role in the formation of typhoid toxin transport intermediates during Salmonella enterica serovar Typhi (S.Typhi) epithelial cell infection.	
Rab11a	P62491	Regulates endocytic recycling. Acts as a major regulator of membrane delivery during cytokinesis. Together with MYO5B and RAB8A participates in epithelial cell polarization. Together with RAB3IP, RAB8A, the exocyst complex, PARD3, PRKCI, ANXA2, CDC42 and DNMBP promotes transcytosis of PODXL to the apical membrane initiation sites (AMIS), apical surface formation and lumenogenesis. Together with MYO5B participates in CFTR trafficking to the plasma membrane and TF (Transferrin) recycling in nonpolarized cells. Required in a complex with MYO5B and RAB11FIP2 for the transport of NPC1L1 to the plasma membrane. Participates in the sorting and basolateral transport of CDH1 from the Golgi apparatus to the plasma membrane. Regulates the recycling of FCGRT (receptor of Fc region of monomeric Ig G) to basolateral membranes. May also play a role in melanosome transport and release from melanocytes.	Plays a role in Golgi-to-ER retrograde trafficking (Galea et al., 2015). Required for regulating cargo sorting at apical recycling endosomes and carrier vesicle docking at the apical membrane (Thuenauer et al., 2014). Transport vesicles containing megalencephalic leukoencephalopathy with subcortical cysts (MLC) along the endosomal recycling pathway (Petrini et al., 2013). Like Rab6a, silencing of Rab11a reverts Golgi fragmentation caused by knockdown of golgin-84 (Heymann et al., 2013). Binding protein FIP1/RCP and required for the retrograde delivery of TGN38 and Shiga toxin from early/recycling endosomes to the TGN (Jing et al., 2010). Interacting with Rab6-interacting protein 1 (R6IP1), and may couple with the function of Rab6 throughout the cell cycle (Miserey-Lenkei et al., 2007). Mediates trafficking of protease-activated receptor 2 from the Golgi apparatus to the plasma membrane (Roosterman et al., 2003).
Rab33b	Q9H082	Protein transport. Acts, in coordination with RAB6A, to regulate intra-Golgi retrograde trafficking. It is involved in autophagy, acting as a modulator of autophagosome formation.	Implicated in Golgi-to-ER retrograde trafficking and maintenance of Golgi ribbon organization (Valsdottir et al., 2001; Starr et al., 2010).
**Suppress COG3 knockdown only**			
None			
(B) Kinesins			
**Suppress both ZW10 and COG3 knockdown**			
Kif25	Q9UIL4	Negative regulator of amino acid starvation-induced autophagy.	No items found.

KifC3	Q9BVG8	Minus-end microtubule-dependent motor protein. Involved in apically targeted transport (By similarity). Required for zonula adherens maintenance.	May play a role in minus-end directed peroxisomal transport (Dietrich et al., 2013). Under the cholesterol-depleted condition, disruption of the kifC3 gene induced fragmentation of the Golgi apparatus, indicating that KifC3 plays a role in Golgi positioning (Xu et al., 2002).
**Suppress ZW10 knockdown only**			
Kif2C	Q99661	In complex with KIF18B, constitutes the major microtubule plus-end depolymerizing activity in mitotic cells. Regulates the turnover of microtubules at the kinetochore and functions in chromosome segregation during mitosis.	No items found.
Kif3A	Q9Y496	Microtubule-based anterograde translocator for membranous organelles. Plus end-directed microtubule sliding activity in vitro. Plays a role in primary cilia formation (By similarity).	HeLa cells depleted of Kif3A by RNAi showed weak dispersal of Golgi membrane markers, indicating that Kif3A is involved in maintaining Golgi structure (Stauber et al., 2006). In addition, Kif3A is also important in intraflagellar transport and Golgi trafficking (Evans et al., 2010; Zhang and Hancock, 2004).
Kif6	Q6ZMV9	NA	No items found.
Kif9	Q9HAQ2	NA	Required for regulation of podosomes in primary human macrophages. The KIF9 C-terminus induces podosome disruption and Golgi dispersal, while depletion of Kif9 by RNAi did not lead to significant alterations in Golgi architecture (Cornfine et al., 2011).
Kif17	Q9P2E2	Transports vesicles containing N-methyl-D-aspartate (NMDA) receptor 2B along microtubules.	Involved in the transport of NMDA receptors from somatic endoplasmic reticulum to a dendritic endoplasmic reticulum subcompartment, and subsequently to Golgi outposts (Jeyifous et al., 2009). Phosphorylation of Kif17 is required for the activity-induced confinement of secretory cargo (Hanus et al., 2014).
KifC2	Q96AC6	May play a role in microtubule-dependent retrograde axonal transport. May function as the motor for the transport of multivesicular body (MVB)-like organelles in dendrites (By similarity).	In neuronal dendrites, expression level of KifC2 was decreased upon chronic alcohol exposure, which caused fragmentation of Golgi apparatus (Romero et al., 2015).

Kif7	Q2M1P5	Essential for hedgehog signaling regulation (PubMed:21633164). Involved in the regulation of microtubular dynamics. Required for proper organization of the ciliary tip and control of ciliary localization of SUFU-GLI2 complexes (By similarity). Required for localization of GLI3 to cilia in response to Shh. Negatively regulates Shh signaling by preventing inappropriate activation of the transcriptional activator GLI2 in the absence of ligand. Positively regulates Shh signaling by preventing the processing of the transcription factor GLI3 into its repressor form. In keratinocytes, promotes the dissociation of SUFU-GLI2 complexes, GLI2 nuclear translocation and Shh signaling activation (By similarity). Involved in the regulation of epidermal differentiation and chondrocyte development (By similarity).	Required for microtubular dynamics. Knockdown of Kif7 with siRNA directed against 3'-UTR of KIF7 mRNA in polarized retinal epithelial cells modified microtubule stability and growth direction, and thus caused defects in cilia formation and induced abnormal centrosomal duplication and fragmentation of the Golgi network (Dafinger et al., 2011).
**Suppress COG3 knockdown only**			
None			

[1]The key words used for searching in PubMed are “Rab, Golgi apparatus” and “Kif, Golgi apparatus”, respectively.
[2]NA (not available) means the information is not showing in UniProt database.

References

Chen T, Han Y, Yang M, Zhang W, Li N, Wan T, Guo J, Cao X. Rab39, a novel Golgi-associated Rab GTPase from human dendritic cells involved in cellular endocytosis. Biochem Biophys Res Commun. 2003; 303(4):1114-1120.

Cornfine S, Himmel M, Kopp P, El Azzouzi K, Wiesner C, Krüger M, Rudel T, Linder S. The kinesin KIF9 and reggie/flotillin proteins regulate matrix degradation by macrophage podosomes. Mol Biol Cell. 2011; 22(2):202-215.

Dafinger C, Liebau MC, Elsayed SM, Hellenbroich Y, Boltshauser E, Korenke GC, Fabretti F, Janecke AR, Ebermann I, Nürnberg G, Nürnberg P, Zentgraf H, Koerber F, Addicks K, Elsobky E, Benzing T, Schermer B, Bolz HJ. Mutations in KIF7 link Joubert syndrome with Sonic Hedgehog signaling and microtubule dynamics. J Clin Invest. 2011; 121(7):2662-2667.

Dietrich D, Seiler F, Essmann F, Dodt G. Identification of the kinesin KifC3 as a new player for positioning of peroxisomes and other organelles in mammalian cells. Biochim Biophys Acta. 2013; 1833(12):3013-3024.

Evans RJ, Schwarz N, Nagel-Wolfrum K, Wolfrum U, Hardcastle AJ, Cheetham ME. The retinitis pigmentosa protein RP2 links pericentriolar vesicle transport between the Golgi and the primary cilium. Hum Mol Genet. 2010; 19(7):1358-1367.

Galea G, Bexiga MG, Panarella A, O'Neill ED, Simpson JC. A high-content screening microscopy approach to dissect the role of Rab proteins in Golgi-to-ER retrograde trafficking. J Cell Sci. 2015; 128(13):2339-2349.

Handley MT, Haynes LP, Burgoyne RD. Differential dynamics of Rab3A and Rab27A on secretory granules. J Cell Sci. 2007; 120(Pt 6):973-984.

Hanus C, Kochen L, Tom Dieck S, Racine V, Sibarita JB, Schuman EM, Ehlers MD. Synaptic control of secretory trafficking in dendrites. Cell Rep. 2014; 7(6):1771-1778.

Heymann J, Rejman Lipinski A, Bauer B, Meyer TF, Heuer D. Chlamydia trachomatis infection prevents front-rear polarity of migrating HeLa cells. Cell Microbiol. 2013; 15(7):1059-1069.

Jeyifous O, Waites CL, Specht CG, Fujisawa S, Schubert M, Lin EI, Marshall J, Aoki C, de Silva T, Montgomery JM, Garner CC, Green WN. SAP97 and CASK mediate sorting of NMDA receptors through a previously unknown secretory pathway. Nat Neurosci. 2009; 12(8):1011-1019.

Jing J, Junutula JR, Wu C, Burden J, Matern H, Peden AA, Prekeris R. FIP1/RCP binding to Golgin-97 regulates retrograde transport from recycling endosomes to the trans-Golgi network. Mol Biol Cell. 2010; 21(17):3041-3053.

Kierszenbaum AL, Tres LL, Rivkin E, Kang-Decker N, van Deursen JM. The acroplaxome is the docking site of Golgi-derived myosin Va/Rab27a/b- containing proacrosomal vesicles in wild-type and Hrb mutant mouse spermatids. Biol Reprod. 2004; 70(5):1400-1410.

Majeed W, Liu S, Storrie B. Distinct sets of Rab6 effectors contribute to ZW10--and COG-dependent Golgi homeostasis. Traffic. 2014; 15(6):630-647.

Miserey-Lenkei S, Waharte F, Boulet A, Cuif MH, Tenza D, El Marjou A, Raposo G, Salamero J, Héliot L, Goud B, Monier S. Rab6-interacting protein 1 links Rab6 and Rab11 function. Traffic. 2007; 8(10):1385-1403.

Petrini S, Minnone G, Coccetti M, Frank C, Aiello C, Cutarelli A, Ambrosini E, Lanciotti A, Brignone MS, D'Oria V, Strippoli R, De Benedetti F, Bertini E, Bracci-Laudiero L. Monocytes and macrophages as biomarkers for the diagnosis of megalencephalic leukoencephalopathy with subcortical cysts. Mol Cell Neurosci. 2013; 56:307-321.

Romero AM, Renau-Piqueras J, Marín MP, Esteban-Pretel G. Chronic alcohol exposure affects the cell components involved in membrane traffic in neuronal dendrites. Neurotox Res. 2015; 27(1):43-54.

Roosterman D, Schmidlin F, Bunnett NW. Rab5a and rab11a mediate agonist-induced trafficking of protease-activated receptor 2. Am J Physiol Cell Physiol. 2003; 284(5):C1319-1329.

Starr T, Sun Y, Wilkins N, Storrie B. Rab33b and Rab6 are functionally overlapping regulators of Golgi homeostasis and trafficking. Traffic. 2010; 11(5):626-636.

Stauber T, Simpson JC, Pepperkok R, Vernos I. A role for kinesin-2 in COPI-dependent recycling between the ER and the Golgi complex. Curr Biol. 2006; 16(22):2245-2251.

Stinchcombe JC, Barral DC, Mules EH, Booth S, Hume AN, Machesky LM, Seabra MC, Griffiths GM. Rab27a is required for regulated secretion in cytotoxic T lymphocytes. J Cell Biol. 2001; 152(4):825-834.

Sun Y, Shestakova A, Hunt L, Sehgal S, Lupashin V, Storrie B. Rab6 regulates both ZW10/RINT-1 and conserved oligomeric Golgi complex-dependent Golgi trafficking and homeostasis. Mol Biol Cell. 2007; 18(10):4129-4142.

Thuenauer R, Hsu YC, Carvajal-Gonzalez JM, Deborde S, Chuang JZ, Römer W, Sonnleitner A, Rodriguez-Boulan E, Sung CH. Four-dimensional live imaging of apical biosynthetic trafficking reveals a post-Golgi sorting role of apical endosomal intermediates. Proc Natl Acad Sci U S A. 2014; 111(11):4127-4132.

Utskarpen A, Slagsvold HH, Iversen TG, Wälchli S, Sandvig K. Transport of ricin from endosomes to the Golgi apparatus is regulated by Rab6A and Rab6A'. Traffic. 2006; 7(6):663-672.

Valsdottir R, Hashimoto H, Ashman K, Koda T, Storrie B, Nilsson T. Identification of rabaptin-5, rabex-5, and GM130 as putative effectors of rab33b, a regulator of retrograde traffic between the Golgi apparatus and ER. FEBS Lett. 2001; 508(2):201-209.

Wang S, Ma Z, Xu X, Wang Z, Sun L, Zhou Y, Lin X, Hong W, Wang T. A role of Rab29 in the integrity of the trans-Golgi network and retrograde trafficking of mannose-6-phosphate receptor. PLoS One. 2014; 9(5):e96242.

Xu Y, Takeda S, Nakata T, Noda Y, Tanaka Y, Hirokawa N. Role of KIFC3 motor protein in Golgi positioning and integration. J Cell Biol. 2002; 158(2):293-303.

Young J, Stauber T, del Nery E, Vernos I, Pepperkok R, Nilsson T. Regulation of microtubule-dependent recycling at the trans-Golgi network by Rab6A and Rab6A'. Mol Biol Cell. 2005; 16(1):162-177.

Zhang Y, Hancock WO. The two motor domains of KIF3A/B coordinate for processive motility and move at different speeds. Biophys J. 2004; 87(3):1795-1804.

Supplemental Figures

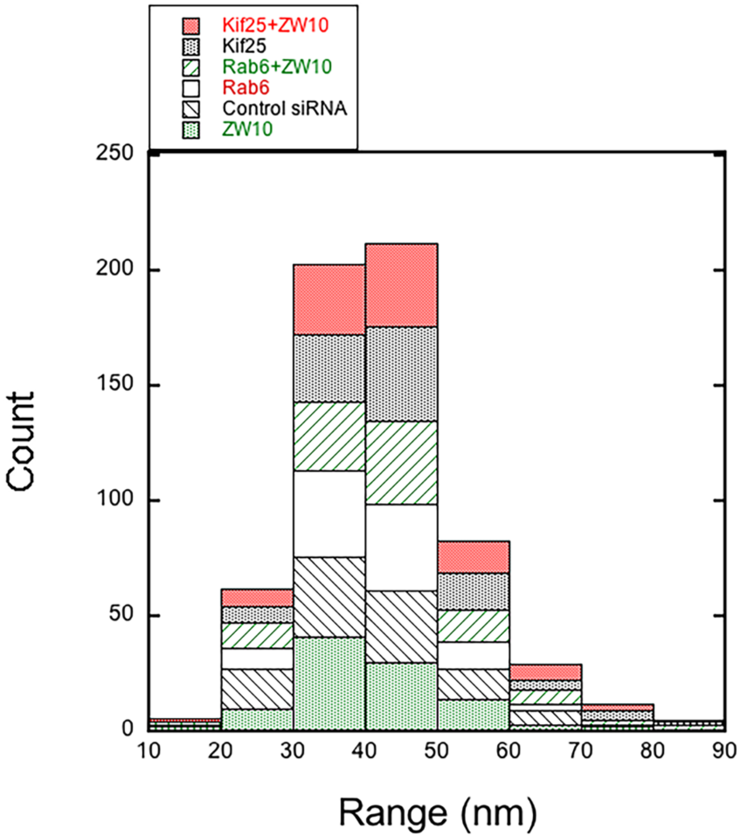

Supplemental Figure 1. Histogram of thin-section size measurements of Golgi-proximal vesicles upon knockdown of Rab6 and Kif25 with/without double knockdown of ZW10. As these measurements are made in thin section, the apparent diameter values indicated in nm are generally an **under estimate** of the actual vesicle diameter as section that cut through the middle of the will reflect the average vesicle diameter. Therefore, the maximal values shown best approximate the actual vesicle diameters. These values are used in the main body of the text.

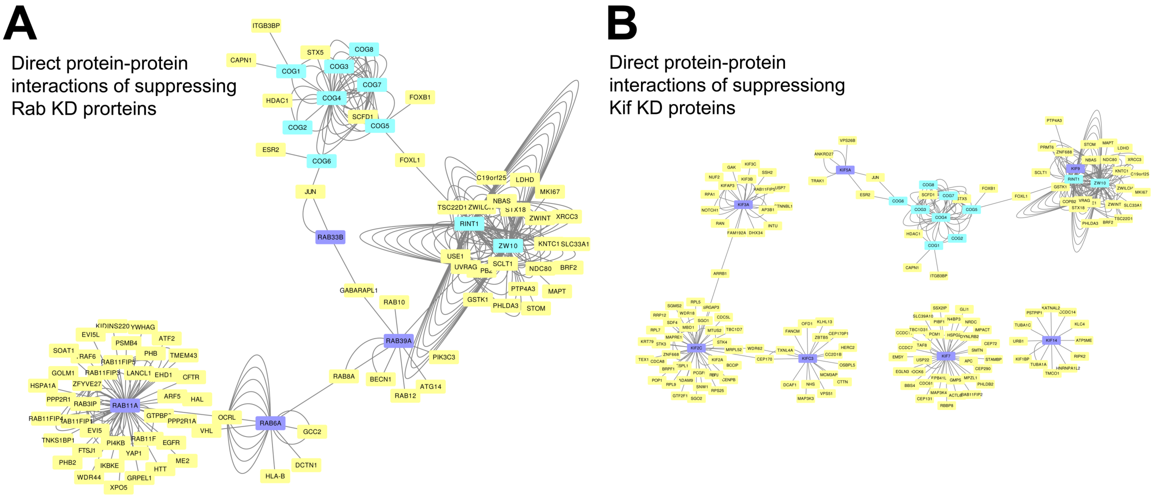

Supplemental Figure 2. Direct protein-protein interactions of Golgi fragmentation-suppressing Rabs (A) and Kifs (B). Protein-protein interaction networks were constructed as described in Materials and Methods. Color code for the nodes: purple, hit Rabs and kinesins; yellow, direct interactors of the screening hits; cyan, proteins of tethering complexes. See also **Supplemental Table 5**, Excel spreadsheets.

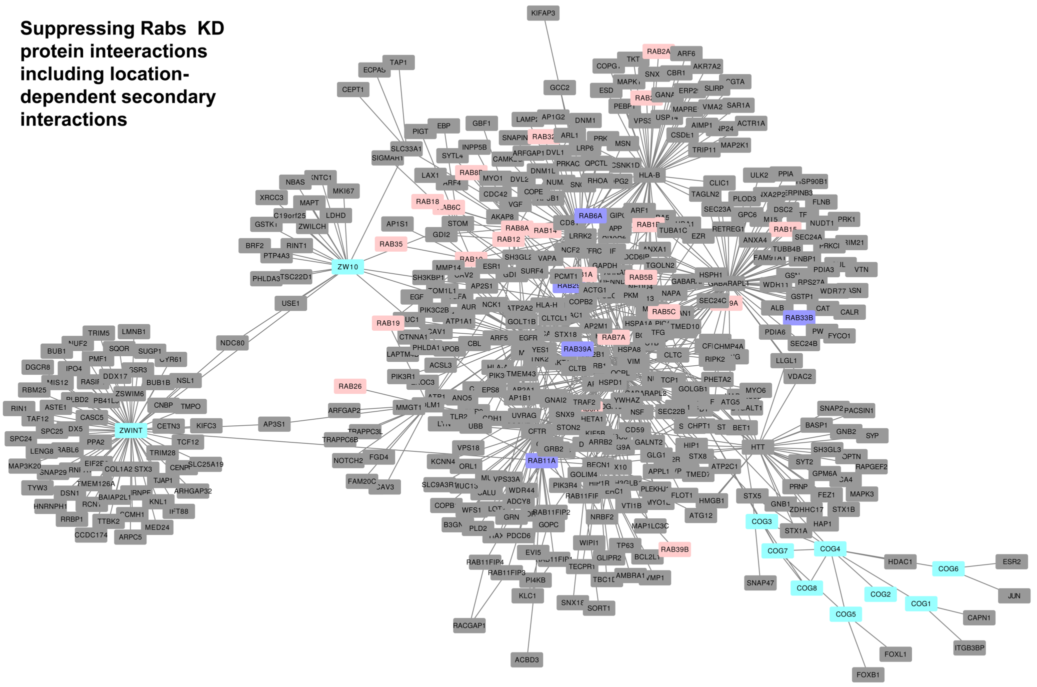

Supplemental Figure 3. Protein-protein interaction network of Golgi fragmentation-suppressing Rabs and their localization-dependent secondary interaction partners. Protein-protein interaction networks were constructed as described in Materials and Methods and for the further analysis, interaction partners with localization annotation of “Golgi” or “vesicle” were considered only. Color code for the nodes: purple, hit Rabs and kinesins; cyan, proteins of tethering complexes; pink, Rabs without a suppressor phenotype; grey, rest of network interaction partners. See also **Supplemental Table 5**, Excel spreadsheets.

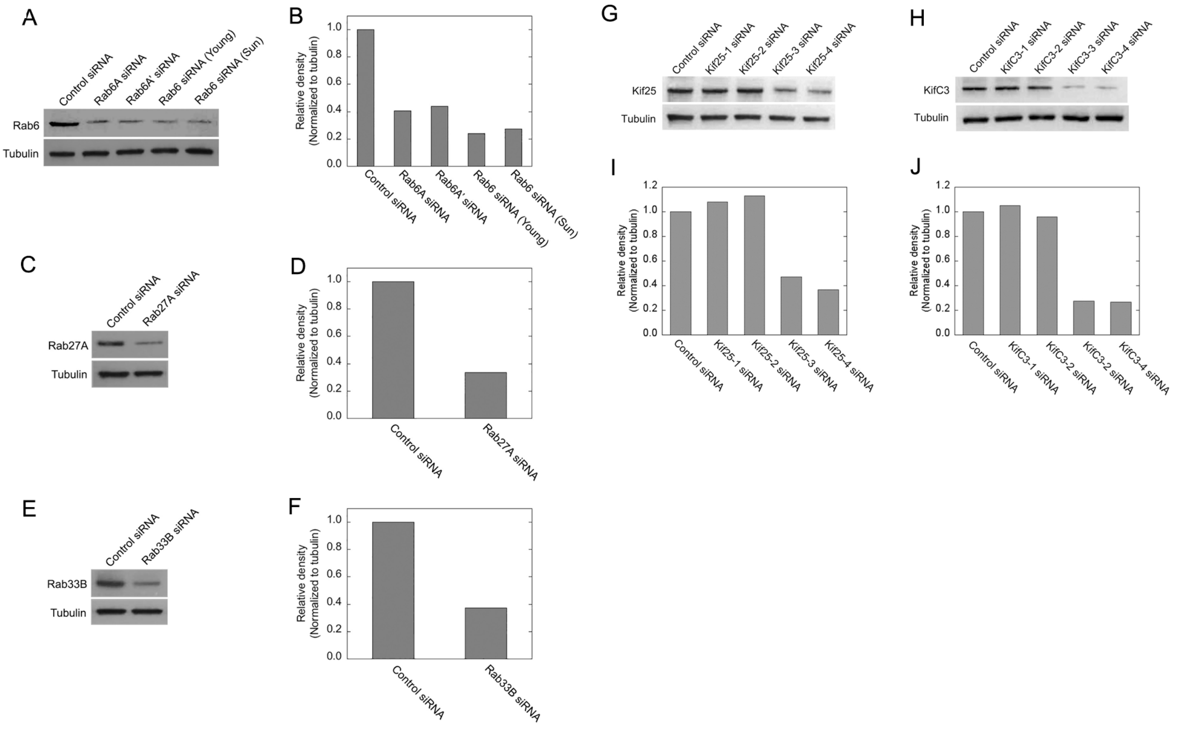

Supplemental Figure 4. **Quantification of Western blot results for select Rabs and Kifs.** A,B) Rab6 knockdowns, C,D) Rab27A, E,F) Rab33B. G,H, I,J) Kif25 and KifC3. 4 day siRNA treatment at 100 nM.
